# Supplementary material for: DNA origami cryptography for secure communication
Source: Nat Commun. 2019 Nov 29;10:5469. doi: 10.1038/s41467-019-13517-3 (PMC6884444; doi:10.1038/s41467-019-13517-3)
Supplement: Supplementary file 1 — Supplementary information [file 41467_2019_13517_MOESM1_ESM.pdf]

Supplementary Information for

DNA origami cryptography for secure communication

Yinan Zhang,[1,2]† Fei Wang,[1]† Jie Chao,[3] Mo Xie,[2] Huajie Liu,[4]* Muchen Pan,[2] Enzo Kopperger,[5] Xiaoguo Liu,[1] Qian Li,[1] Jiye Shi,[2] Lihua Wang,[2,6] Jun Hu,[2] Lianhui Wang,[3] Friedrich C. Simmel,[5] and Chunhai Fan[1]*

[1]School of Chemistry and Chemical Engineering, and Institute of Molecular Medicine, Renji Hospital, School of Medicine, Shanghai Jiao Tong University, Shanghai 200240, China.

[2]Division of Physical Biology, CAS Key Laboratory of Interfacial Physics and Technology, Shanghai Institute of Applied Physics, Chinese Academy of Sciences, Shanghai 201800, China.

[3]Key Laboratory for Organic Electronics & Information Displays (KLOEID), Institute of Advanced Materials (IAM) and School of Materials Science and Engineering, Nanjing University of Posts & Telecommunications, 9 Wenyuan Road, Nanjing 210046, China.

[4]School of Chemical Science and Engineering, Tongji Univeristy, Shanghai, China.

[5]Physics of Synthetic Biological Systems (E14), Physics Department, Technische Universität München, Am Coulombwall 4a, 85748 Garching, Germany.

[6]Shanghai Key Laboratory of Green Chemistry and Chemical Processes, School of Chemistry and Molecular Engineering, East China Normal University, 500 Dongchuan Road, Shanghai, 200241, China.

[7]Shanghai Synchrotron Radiation Facility, Zhangjiang Laboratory, Shanghai Advanced Research Institute, Chinese Academy of Sciences, Shanghai 201210, China.

†The authors contributed equally to this work.

*Correspondence to: liuhuajie@tongji.edu.cn; fanchunhai@sjtu.edu.cn

This PDF file includes:

Supplementary Methods
Supplementary Discussion
Supplementary Figs. 1 to 31
Supplementary Notes: DNA sequences
Supplementary References

Supplementary Methods

Materials. Biotinylated DNA strands purified by HPLC were purchased from Sangon Biotechnology. Alexa 647-labeled DNA strands purified by HPLC were purchased from Invitrogen. Unmodified DNA strands purified by PAGE were purchased form JieLi Biology. Streptavidin was purchased from Sigma-Aldrich. M13mp18 scaffold strands were purchased from New England Biolabs. FluoSphere™ carboxylate-modified microspheres were purchased from Thermo Fisher. All other chemicals were purchased from Sinopharm. Water was purified with a Millipore Milli-Q Integral water purification system (resistivity=18.2 MΩ·cm).

Choosing an appropriate scaffold. A geometry that covered the spot pattern conveying the message was firstly defined. Secondly, the scaffold length fitting the defined geometry in a raster-filling method was determined. A short scaffold (below 150 nt) can be synthesized chemically. Fabrication of longer scaffold can be achieved from a natural plasmid template. The scaffold used here, M13mp18, was commercially achievable.

Generating sequences of M- and staple strands. Sequences of M- and staple strands depended on the scaffold of DNA origami. Previously, the scaffold was folded back and forth to fill the defined geometry covering the spot pattern, revealing the correspondence between M-strands and spots. Therefore, the sequence of M-strands at individual spots were generated from the scaffold. Sequences of staple strands can be generated based on the scaffold folding with aid of professional software such as caDNAno. However, a DNA origami design not restricted to classical models needs to be finished manually. It should be noted that the M-strands hybridized with the scaffold are forbidden to hinder scaffold crossovers in DNA origami. Nevertheless, some staple crossovers are inevitably hindered by the spanning M-strands. Elaborate adjustment was performed to arrange M-strands on the scaffold at minimum sacrifice of staple crossovers. No evident damage caused by loss of local staple crossovers to DNA origami was observed in the experiment.

Binding M-strands to DNA scaffold. 200 nM M-strands were mixed with 20 nM scaffold strands in 1×TAE buffer (40 mM Tris, 20 mM acetic acid, 2 mM EDTA, pH 8.0) with 12.5 mM Mg^{2+}. Excess M-strands facilitated a complete hybridization with the scaffold. A rapid anneal from 85 ºC to 4 ºC was then performed. Afterwards, unbound M-strands were removed by centrifuge filters. The molecular weight cut-off (MWCO) of centrifuge filters depends on the length of DNA scaffold. 100 kDa is ideal for M13mp18 scaffold.

Delivery of DNA scaffold. Scaffold strands carrying different M-strands can be collected in tube and directly delivered to Bob. Alternatively, the collected mixture can be dropped onto a paper for delivery. The dropped spot on the paper was cut and soaked in 1×TAE buffer with 12.5 mM Mg^{2+} for 30 min. After that, the remnant was squeezed and the supernatant was collected. Fresh buffer was then added to rinse the remnant. After three times of rinsing, the collected supernatant was concentrated to a final concentration of 20 nM.

DNA origami folding. Staple strands were preheated to 95 ºC for 3 min and cooled to room temperature slowly. Scaffold strands carrying M-strands were then mixed with the staples at a molar ratio of 1:10 in 1×TAE buffer with 12.5 mM Mg^{2+}. The final concentration of scaffold

strands was maintained at 2 nM. Afterward, the mixture was heated at 57 ºC for 3 min and then annealed to 27 ºC at a rate of -5 ºC min^{-1}. Folded DNA origami was then purified with 100 kDa (MWCO) centrifuge filters three times to remove excess M- and staple strands.

Joining E- and F- tiles. Linker strands were mixed with E- and F-tiles at ten times the concentration of the sticky ends. The mixture was then annealed slowly from 45 ºC to 25 ºC in three cycles and finally held at 25 ºC.

Adding streptavidin to recognize biotin patterns. Streptavidin was added to recognize the biotin patterns on DNA origami at a molar ratio of 10:1 to the biotin on DNA origami. After a 2-hour incubation at room temperature, the patterns were characterized under the AFM.

AFM imaging. A droplet (~2 μL) was deposited on freshly cleaved mica surface and left to absorb for 3 min. After that 40 μL of 1×TAE buffer containing 12.5 mM Mg^{2+} was added to the liquid cell and a NP-S (Bruker, Inc.) tip was used to scan the sample in a PeakForce-tapping mode on a Multimode VIII AFM (Bruker, Inc.). A minimum force was maintained in imaging to prevent scratching of streptavidin by the tip which could led to false negative results. DNA origami showed a high tendency to aggregate in 1×TAE buffer containing 12.5 mM Mg^{2+}. Removal of staples strands binding at the edge of DNA origami from the staple library alleviated the aggregation. Nevertheless, AFM characterization should be undertaken soon after the addition of streptavidin to DNA origami. Undistinguishable patterns on aggregation of DNA origamis were excluded from statistics.

STORM imaging. Alexa 647-labeled DNA strands were added at ten times the concentration of the anchors on DNA origami. After an overnight incubation at 25 ºC, free strands were removed with 100 kDa (MWCO) centrifuge filters. The DNA origami was dropped on a glass dish at a concentration of ~100 pM. Before the deposition, the glass dish was treated with negative glow discharge. FluoSphere™ carboxylate-modified microspheres were used as the drift marker. Imaging was performed with inclined illumination at an excitation intensity of 200 W cm^{-2} at 488 nm and 647 nm. Images were reconstructed from more than 30000 frames at an interval of 20 ms. ImageJ was used for image processing with Gaussian fitting algorithms.

Supplementary Discussion

1. Modeling the dissociation of M-strands from DNA scaffold

The disassociation of M-strands from the scaffold during DNA origami folding was modeled with NUPACK (www.nupack.org/) (Supplementary Figure 4). Four DNA segments (from A to D) hybridized to different sites of the scaffold that distributed them among the four corners of DNA origami were involved in the investigation. Three lengths, 32, 40 and 48 nucleotides, were accounted for each segment. The concentrations of DNA, Na^+ and Mg^{2+} were set at 2 nM, 50 mM (the lower limit for Na^+ in NUPACK) and 12.5 mM, respectively. The temperature ranged from 51 ºC to 61 ºC with an increment of 2 ºC. Though there was no Na^+ in the 1×TAE buffer used, the effect of 50 mM Na^+ on binding stability of segments was negligible compared to that of 12.5 mM Mg^{2+}. Hence, the simulation literally reflects the stability of DNA segments of different lengths binding on the scaffold in the buffer when the temperature was elevated. Fractions of bases unpaired in every DNA segment from 51 ºC to 61 ºC for all the

groups were compared. The 40-nt group in the four segments shows fairly low fractions of bases unpaired (A: 5.4%; B: 4.8%; C: 7.6%; D: 8.7%) at 57 ºC in the buffer (the starting temperature of anneal, T_A). The unpaired fractions for the 32-nt group are higher than the 40-nt group except in C where both groups show a robust binding. The 48-nt group binds slightly more stable but cause further loss of staple crossovers. Low unpaired fractions and loss of staple crossovers are appreciated in determining the lengths for individual segments. Accordingly, the 40-nt length is chosen for DNA segments in A and B while the 48-nt length is chosen in C and D to keep M-strands bound to the scaffold during the folding of DNA origami.

2. Sequencing the intercepted scaffold

DNA media conveying the message can be transmitted in a very concealed way. Nevertheless, we assume Mallory manages to intercept the media and sequence the scaffold. Though the primers for sequencing are not given, a plausible method is sketched here (Supplementary Figure 7). It's literally the most convenient one as we know.

The method consists of three steps, DNA double strand formation, DNA amplification and sequence splice. The former step resembles a reverse transcription process. Firstly, a pool of random primers (heptaribonucleotides, 100 pmol for μg^{-1} DNA scaffold) is introduced to hybridize with the DNA scaffold at 65 ºC for 5 min, followed by an incubation at ice bath for 1 min. Reverse transcriptase, dNTPs and RNAse inhibitors are then added to elongate the primers at room temperature for 10 min. Subsequently, the reverse transcriptase is deactivated by heating at 70 ºC for 10 min, after which a pool of DNA double strands is prepared.

The second step is a polymerase chain reaction (PCR) with tagged random primers[1-2] which are comprised of a 9 base pairs (bp) arbitrary 3' tail that bind anywhere on the DNA double strands and a constant 17 bp 5' head for second-round amplification. The tagged random primers are initially annealed with DNA double strands at 30 ºC for 1 min to bind their 3' tail on complementary segments after denaturation at 96 ºC for 30 secs. The mass ratio of primers to samples is maintained at ~30. DNA polymerase and dNTPs are next introduced to elongate the primers at 72 ºC for 2 min. The cycles are repeated three times, producing DNA copies with tagged random primers attached at their ends. After free tagged random primers and their complexes are removed, a second-round amplification using a single sequence-specific primer which matches the 5' head of tagged random primers is performed. The concentration of the single primers is 2.5 μM. The temperatures during the second-round amplification follow the same with the first one except that the single primer is annealed with DNA double strands at 55 ºC. 30 cycles are repeated, followed by recovery of the product for sequencing. Different fragments of the scaffold can be sequenced, which requires a splice to read the complete sequence of the double-strand scaffold. The sequence of single-strand scaffold can be either one in the reading result of the double strands. In turn, the key size derived from the sequence and length of DNA scaffold could be reduced to one bit (2^1).

3. Calculating the key size of DOE

A model decomposing the folding of scaffold into two factors, the sliding and routing of scaffold in DNA origami, was built to approximately quantity the key size of DNA origami encryption for Mallory (Supplementary Figure 8). The scaffold is folded in a raster-filled way, resulting in individual DNA origami bundles parallelly aligned from top to bottom row. Each bundle has a length of a multiple of 10.5 nucleotides which is exactly the pitch of B-DNA helix to lessen internal tension in a DNA origami. Commonly used scaffolds like M13mp18 have a

length of over 7000 nucleotides with a fraction of them not actually folded by staple strands in DNA origami, which adds to difficulty for Mallory. For convenience we assume every nucleotide of DNA scaffold has been paired in DNA origami, which implies that the sum length of the bundles is identical to that of the scaffold strand. Routing the scaffold strand is essentially parting it into individual bundles, which is equal to an integer composition problem that parts a positive integer *n* into the sum of a sequence of positive integers. Let *k* be the number of rows of bundles, the total number of compositions of *n* is calculated by

$$N_1 = \sum_{k=1}^{n} \binom{n-1}{k-1} = 2^{n-1}. \tag{1}$$

The value of *n* is given by

$$n = \left\lfloor \frac{L}{10.5} \right\rfloor. \tag{2}$$

It's noted that when a certain bundle of DNA origami is extremely long (for example, it has the same length as the scaffold), the DNA origami could be squeezed into a double strand. However, this situation is acceptable since biotin arranged on a long double strand can still convey some message. Scaffold sliding in DNA origami, which determines the starting point and direction of sequence assignment from a routing-defined scaffold to staple strands, is another factor that induces variation of the biotin pattern. It can be described by

$$N_2 = L, \tag{3}$$

where *L* is the length of the scaffold. In consequence, the key size of DNA origami encryption for Mallory is given by

$$K_{DOE}^{M} = \log_2(2N_1N_2), \tag{4}$$

when combined with Eq. S1 and S3 can be further reduced as

$$K_{DOE}^{M} = \left\lfloor \frac{L}{10.5} \right\rfloor + \log_2 L. \tag{5}$$

The factor "2" in Eq. S4 derives from the DNA scaffold sequencing as described above. From Eq. S5 we find that there is a positive correlation between the length of the scaffold strand and the key size of DNA origami encryption for Mallory. The key size reaches 702 bits for a 7249-nucleotide M13mp18 scaffold, which has a significant advantage over AES with a key size no more than 256 bits. It should be noted that some situations excluded from the model could happen in practice:

a. Portions of the scaffold remain in loops instead of folding;

b. More than one bundle could stand in the same row;

c. Some bundles could be nonparallel to the others.

Each situation could induce more difficulty for Mallory, which makes the encryption stronger in practice.

4. Brute-force attack

On the scaffold strand:

The calculation of key size for a brute-force attack on the scaffold strand resembles that for Mallory except that a sum of available lengths of scaffold strands are considered for counting N_0, N_1 and N_2:

$$N_0 = \sum_{h=1}^{H} 4^{L_h}, \tag{6}$$

$$N_1 = \sum_{h=1}^{H} 2^{\left\lfloor \frac{L_h}{10.5} \right\rfloor - 1}, \quad (7)$$

$$N_2 = \sum_{h=1}^{H} 2L_h, \quad (8)$$

where H denotes the number of possible lengths of scaffold strands. Hence, the key size for a brute-force attack on scaffold strands is given by

$$K_{DOE}^{BF1} = \log_2 \sum_{h=1}^{H} 4^{L_h} + \log_2 \sum_{h=1}^{H} 2^{\left\lfloor \frac{L_h}{10.5} \right\rfloor} + \log_2 \sum_{h=1}^{H} L_h. \quad (9)$$

On the staple strands:

The distribution of lengths of staple strands as well as their sequences are completely unknow for a brute-force-attacker. Let p_g be the number of staple strands with a length of q_g and L_h be the length of corresponding scaffold, assume the scaffold is fully folded in DNA origami, L_h can be calculated as

$$L_h = \sum_{g=1}^{G} p_g q_g, \quad (10)$$

where G is the number of different lengths of staple strands. Therefore, the distribution of the length of scaffold among the staple strands can be simplified into to a partition problem that parts a positive integer n into a sum of positive numbers. There have been programs to calculate $p(n)$ for a specified a with different methods such as generating function and pentagonal number theorem. However, the value of n is not available in a brute-force attack. For example, n is 690 and 503 for M13mp18 and phi X 174 scaffold, respectively (Eq. 2). The factor concerning the distribution of lengths of staple strands is given by

$$N_3 = \sum_{h=1}^{H} p\left(\left\lfloor \frac{L_h}{10.5} \right\rfloor\right). \quad (11)$$

Besides, the randomness derived from the sequences of staple strands is given by

$$N_4 = \sum_{h=1}^{H} 4^{L_h}. \quad (12)$$

N_4 is equal to N_0 since the scaffold is assumed to be fully folded. The design size of k_{DOE} is described by

$$K_{DOE}^{BF2} = \log_2(N_3 N_4), \quad (13)$$

when combined with Eq. S11 and S12 can be reduced as

$$K_{DOE}^{BF2} = \log_2 \sum_{h=1}^{H} p\left(\left\lfloor \frac{L_h}{10.5} \right\rfloor\right) + \log_2 \sum_{h=1}^{H} 4^{L_h}. \quad (14)$$

DNA origamis with scaffolds of different lengths from several hundred to over twenty thousand have been developed, which makes both L_h as well as H imponderable. Therefore, it's impractical to even quantify the size of k_{DOE} in a brute-force attack.

5. Generation and collision-resistance of the hash value to Bob and Mallory

Each streptavidin pattern comprising the message contains a region (in purple) that represents a digit corresponding to an alphabetical letter from A to G (Supplementary Figure 22). The letters in streptavidin patterns for Bob are from B to G while the letters for Mallory are C, E

and F. With the random dimerization of E- and F-tiles, the hash value of the message is generated as successive permutations of letters from the letter pool. The hash value of message available to Bob or Mallory is:
Bob: GF GG GE GC GB GD BF BG BE BC BB BD EF EG EE EC EB ED DF DG DE DC DB DD CF CG CE CC CB CD FF FG FE FC FB FD;
Mallory: CC CE CF EC EE EF FC FE FF.

Collision-resistance of the hash algorithm for the message is then discussed. Let u be the number of spots representing alphabetical letters used in the algorithm and v denote the kinds of patterns on E- or F- tiles. The hash value pool has a capacity of

$$V = (2^u)^v (2^u)^v = 2^{2uv}. \tag{15}$$

The volume reaches about 2^{36} and 2^{18} for Bob and Mallory respectively, which is practically resilient to collision.

6. Calculation of the yield of tile dimers for Bob and Mallory

The yield of tile dimers was calculated with the equation

$$Y = \frac{2x_1}{(2x_1 + x_2)\cdot z}, \tag{16}$$

where z represents the percentage of tiles carrying certain sticky ends (z=2/3 for Bob and 1/3 for Mallory), x_1 and x_2 is number of E-F tile dimers and monomers, respectively.

7. Password strength for Bob and Mallory

The strength of Bob's and Mallory's password is given by

$$H_{B,M} = \log_2 (4^r)^t = 2rt, \tag{17}$$

where r is the length of every sticky end, and t is the number of sticky ends. Since there are four pairs of eight-nucleotide sequences, the password strength reaches 128 bits.

8. Discussion on "Bit Error Probability".

The "Bit Error Probability" may be reflected in this work by the accuracy of the spot pattern at each position in AFM imaging. We have conducted multiple tests with message of different lengths as well as two blind tests (Supplementary Figures 17-20). We found that in all tests the major patterns that took up over 50% of the population were the correct ones, but there also was a minority of wrong patterns. The error ratio was relevant to the number of biotinylated spots in corresponding positions since the efficiency of conjugation between biotin and streptavidin is only ~95%[3]. Besides, we found that the spot pattern with fewer position spots (spots that indicate the position of the letter) tended to have a higher error ratio. We think this is because once the position spot in another spot pattern is missing, the spot pattern will be wrongly counted in the statistics of the one with the corresponding position spots.

Hence, the bit error ratio is dependent on the number of spots and their distribution in denoting the letters or the positions and thus is different at each position. For example, from the statistics of the message "19120623", we estimated the Bit Error Probability of each letter as follows:

1st "1": 42.4%
2nd "9": 27.7%

3rd "1": 34.7%
4th "2": 32.2%
5th "0": 39.3%
6th "6": 20.6%
7th "2": 32.7%
8th "3": 25.2%

We anticipate that technological advances and new characterization methods will further reduce the bit error probability, so this will not be a fundamental obstacle.

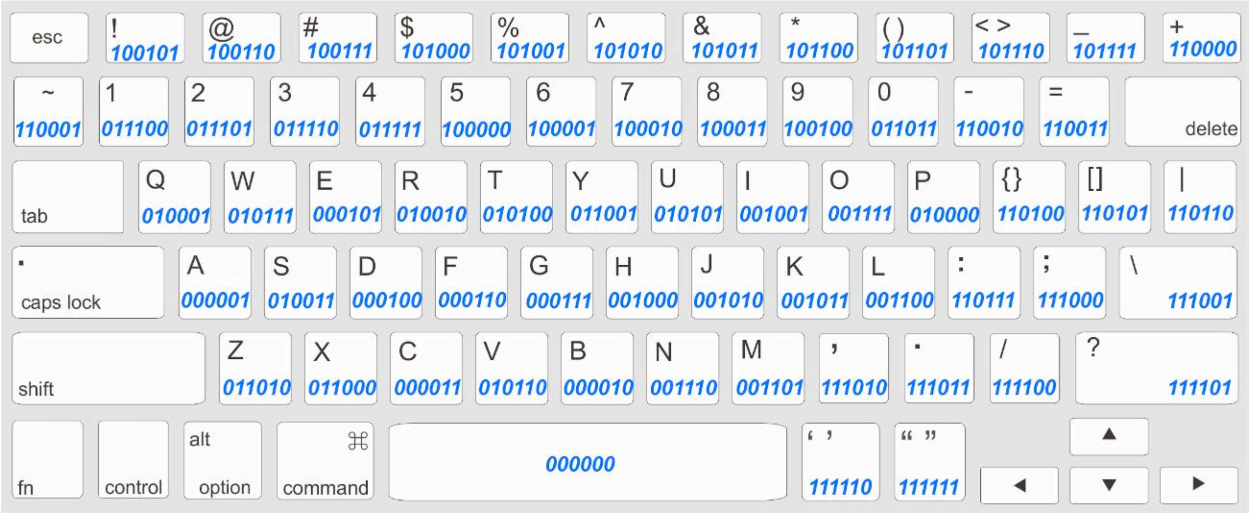

Supplementary Figure 1 | The custom keypad indexing individual letters to binary numbers.

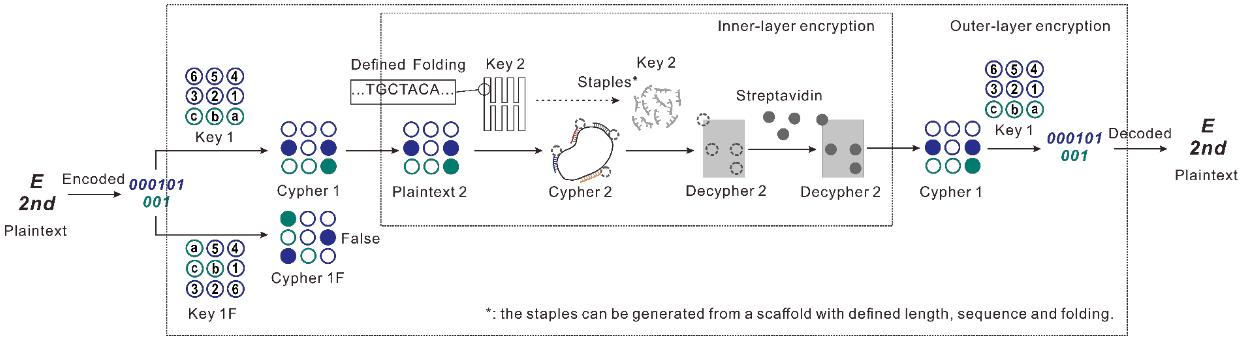

Supplementary Figure 2 | Transfer of an individual letter in the two-layer encryption using shared keys. As is shown above, the first step using a shared key is the encryption of binary numbers into spot patterns in the outer-layer encryption. The key here is the permutation of the spot representing the binary digits of the letters or their positions. The second step using a shared key is the encryption of spot pattern into a scaffold (a long single-stranded viral DNA) carrying certain biotinylated M-strands. The key here is the sequence, length and folding of the long scaffold strand. Besides, a keypad is used to encode the letters into binary numbers. And the intrinsic addressability of DNA origami allows for streptavidin-binding-based steganography. According to Kerckhoffs's principle, only the keys are required to be kept secret.

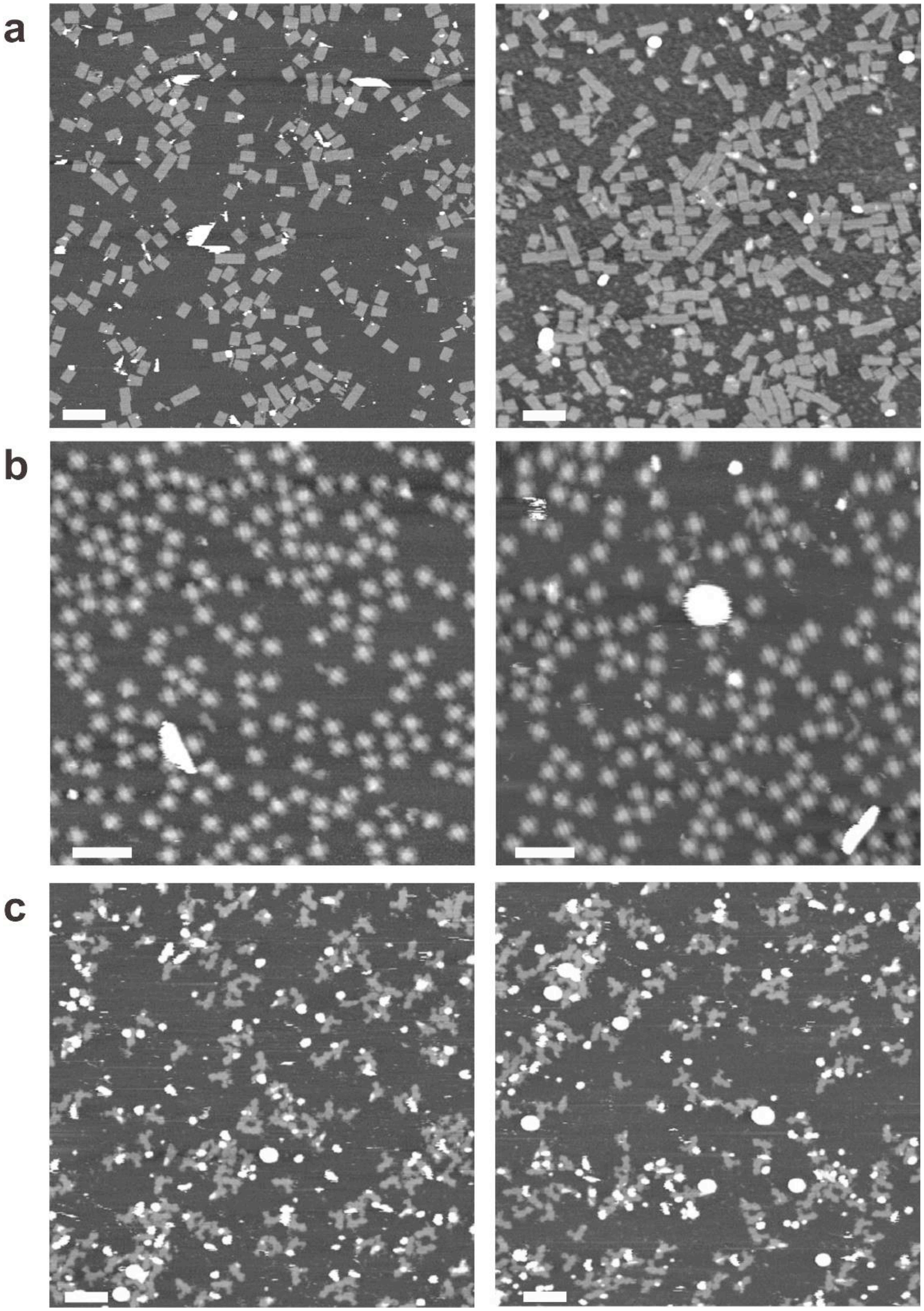

Supplementary Figure 3 | Comparison between rectangular (A), cross-shaped (B) and China map-shaped (C) DNA origamis folded through Rothemund's protocols (left) and anneal from 57 ºC (right). Scale bar: 200 nm.

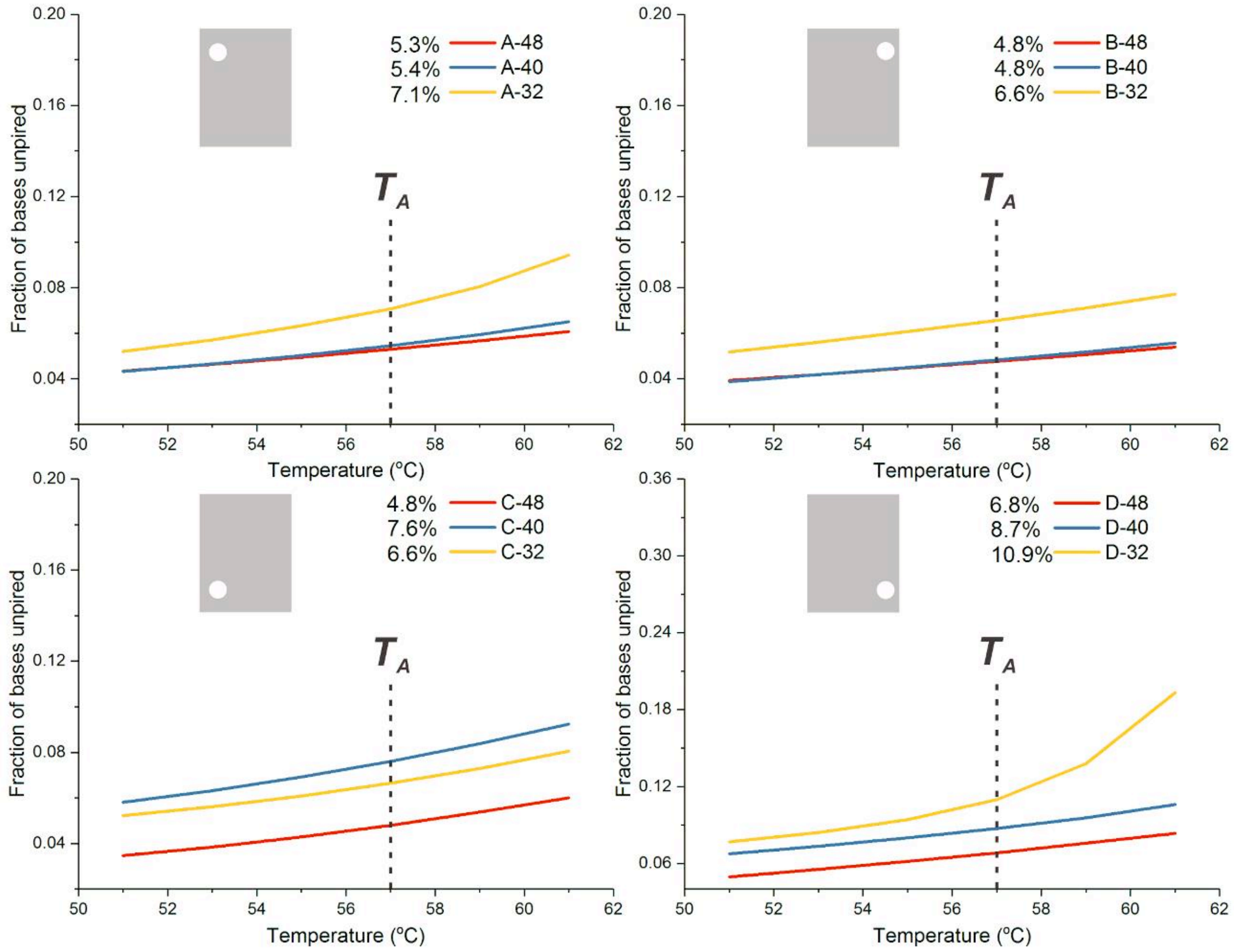

Supplementary Figure 4 | Simulation of the disassociation of DNA segments from the scaffold strand with NUPACK (www.nupack.org/). Fraction of bases unpaired at the starting temperature of anneal, T_A (the dashed line) is given.

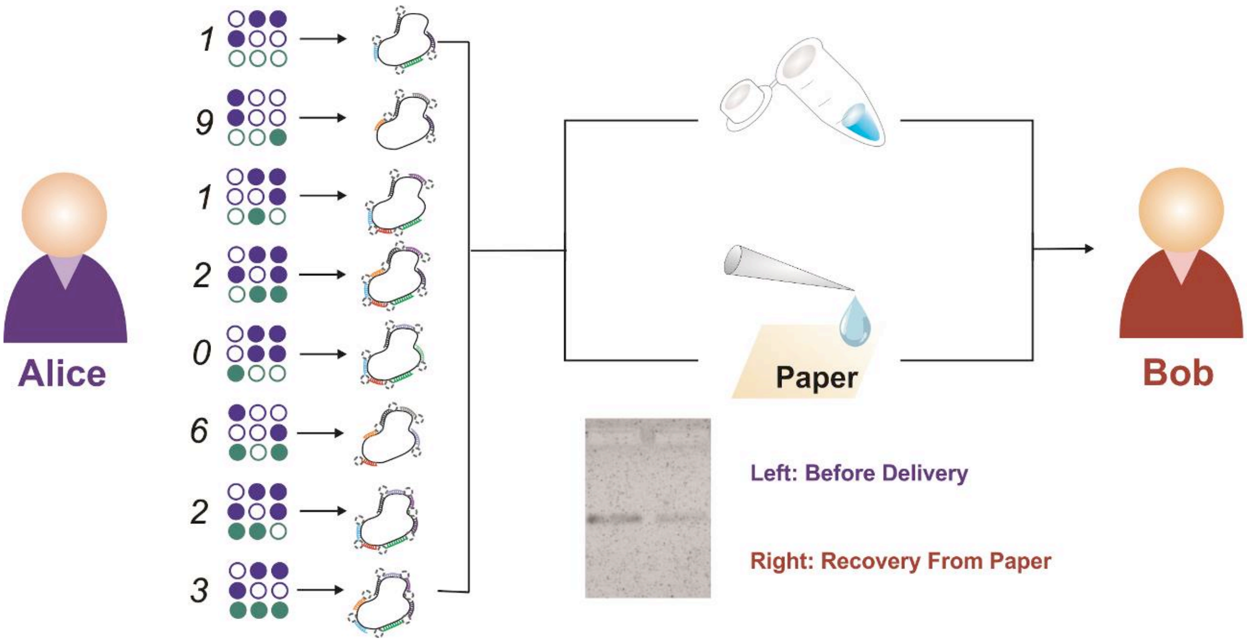

Supplementary Figure 5 | Delivery of encrypted patterns from Alice to Bob. Scaffold strands carrying different M-strands can be collected in tube and directly delivered to Bob. Alternatively, the collected mixture can be dropped on a paper for Bob. The recovered scaffold strands from paper showed a yield at ~53% estimated from the 0.5% gel.

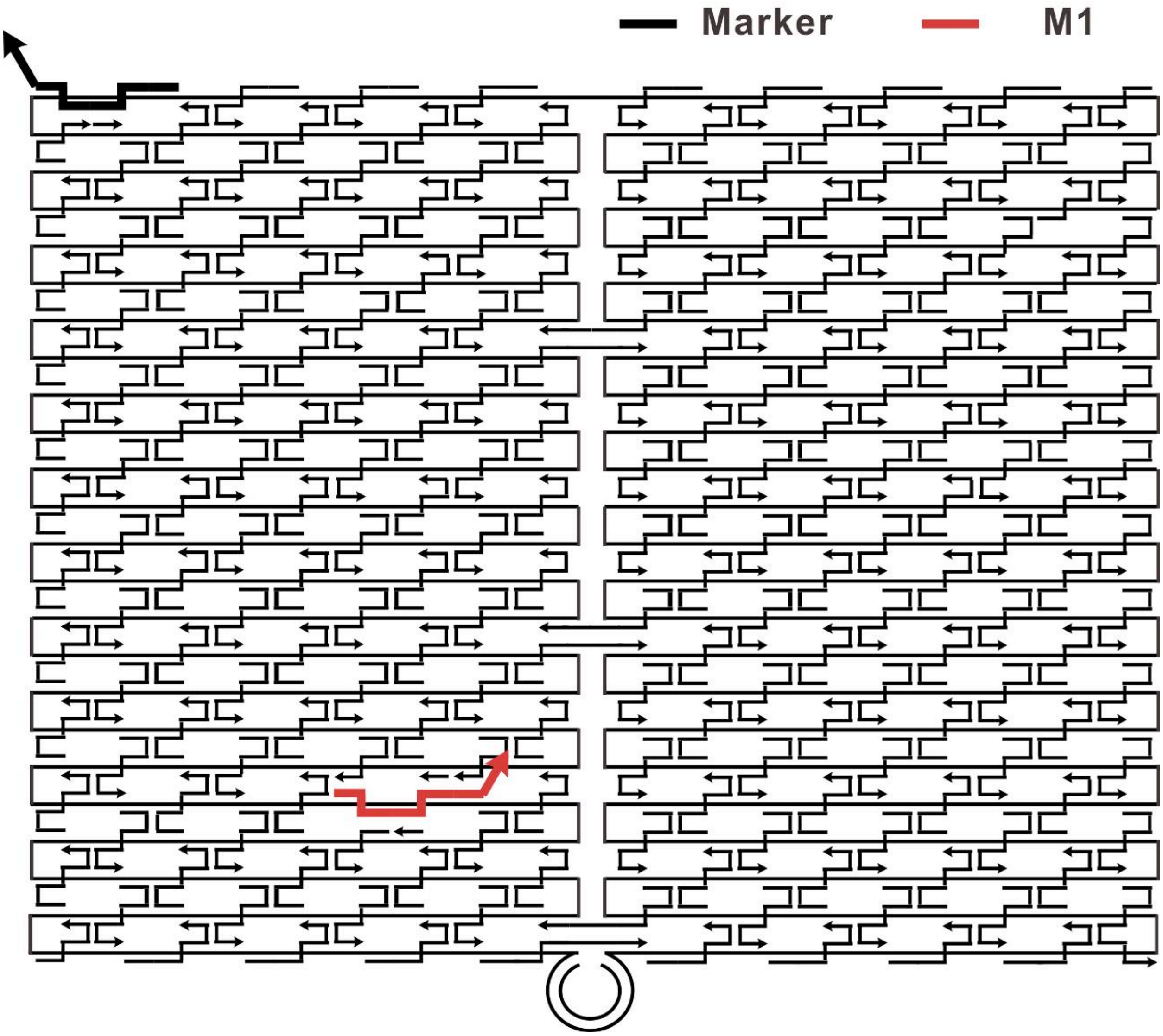

Supplementary Figure 6 | Rectangular DNA origami carrying the strands for the fluorescent pattern. Binding of messages strands along the scaffold inevitably sacrifices some staple crossovers. Unbound sections of staple strands are not depicted.

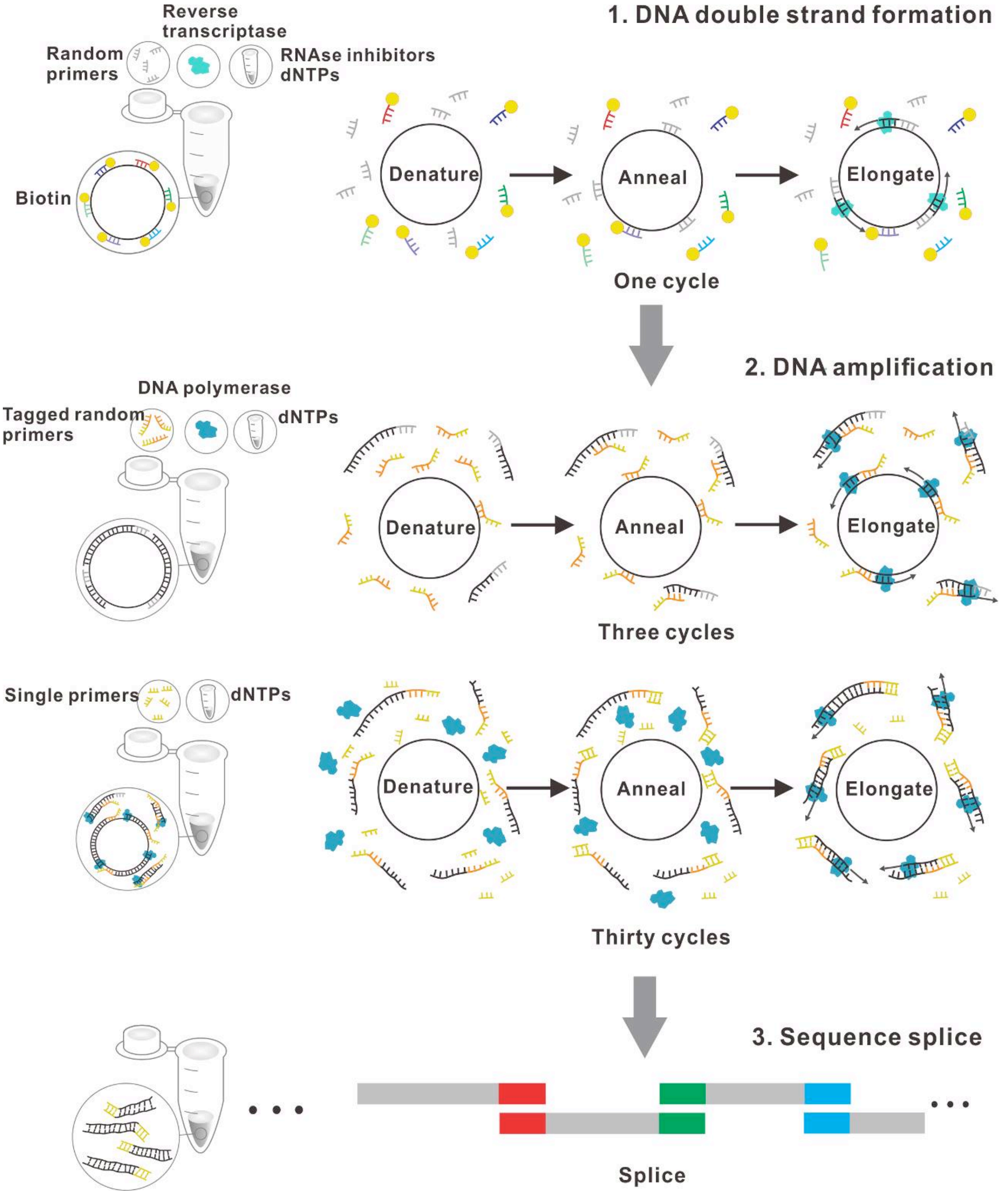

Supplementary Figure 7 | Workflow to sequence the intercepted scaffold. The scaffold is firstly turned into double strand by reverse transcription and then amplified with tagged random primers. A library of DNA double strands contained in the scaffold double strands are produced. Sequencing results of the library are spliced to find out the sequence of the scaffold double strands.

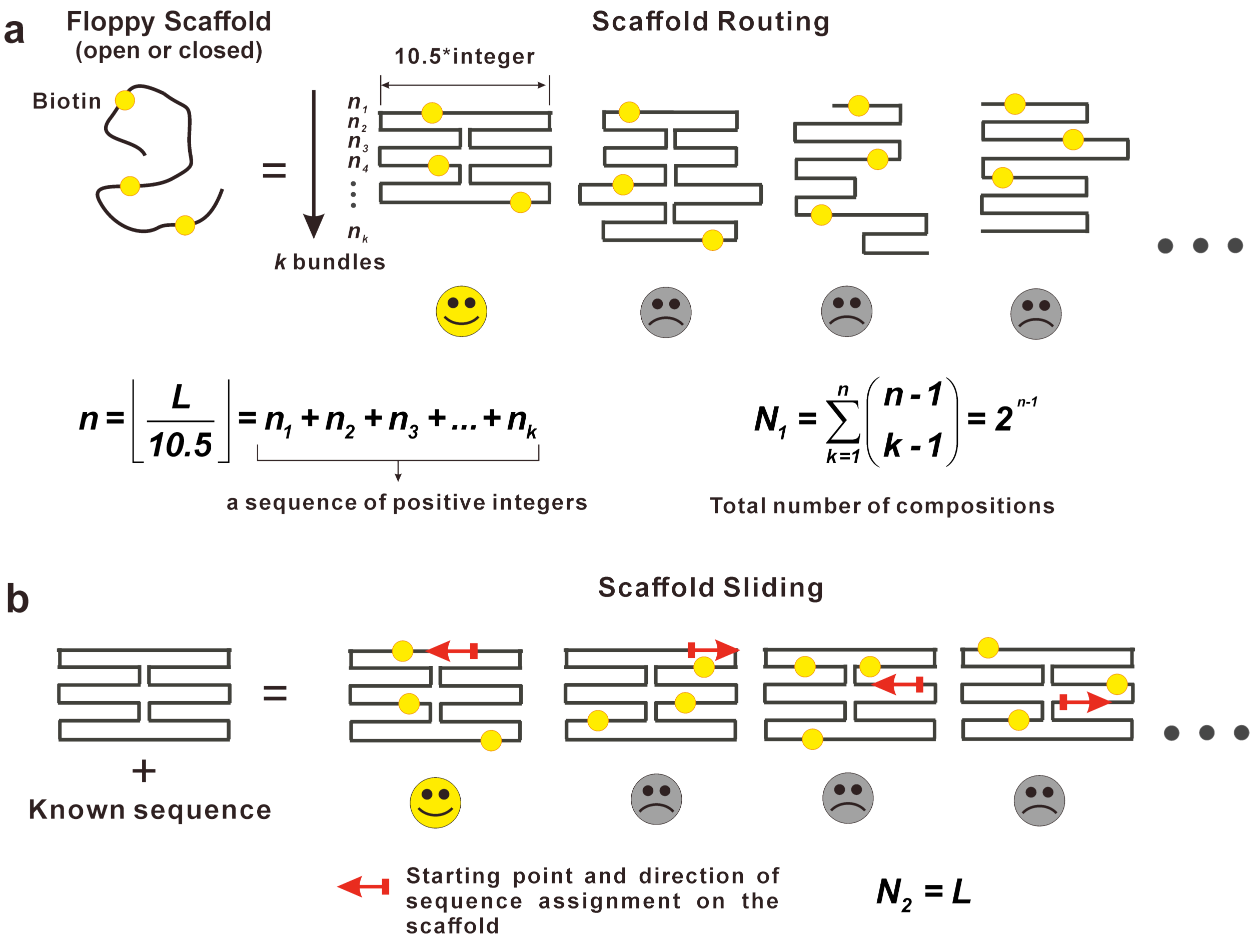

Supplementary Figure 8 | A model concerning routing (a) and sliding (b) of scaffold strand in DNA origami to approximately quantity the key size of DNA origami encryption for Mallory.

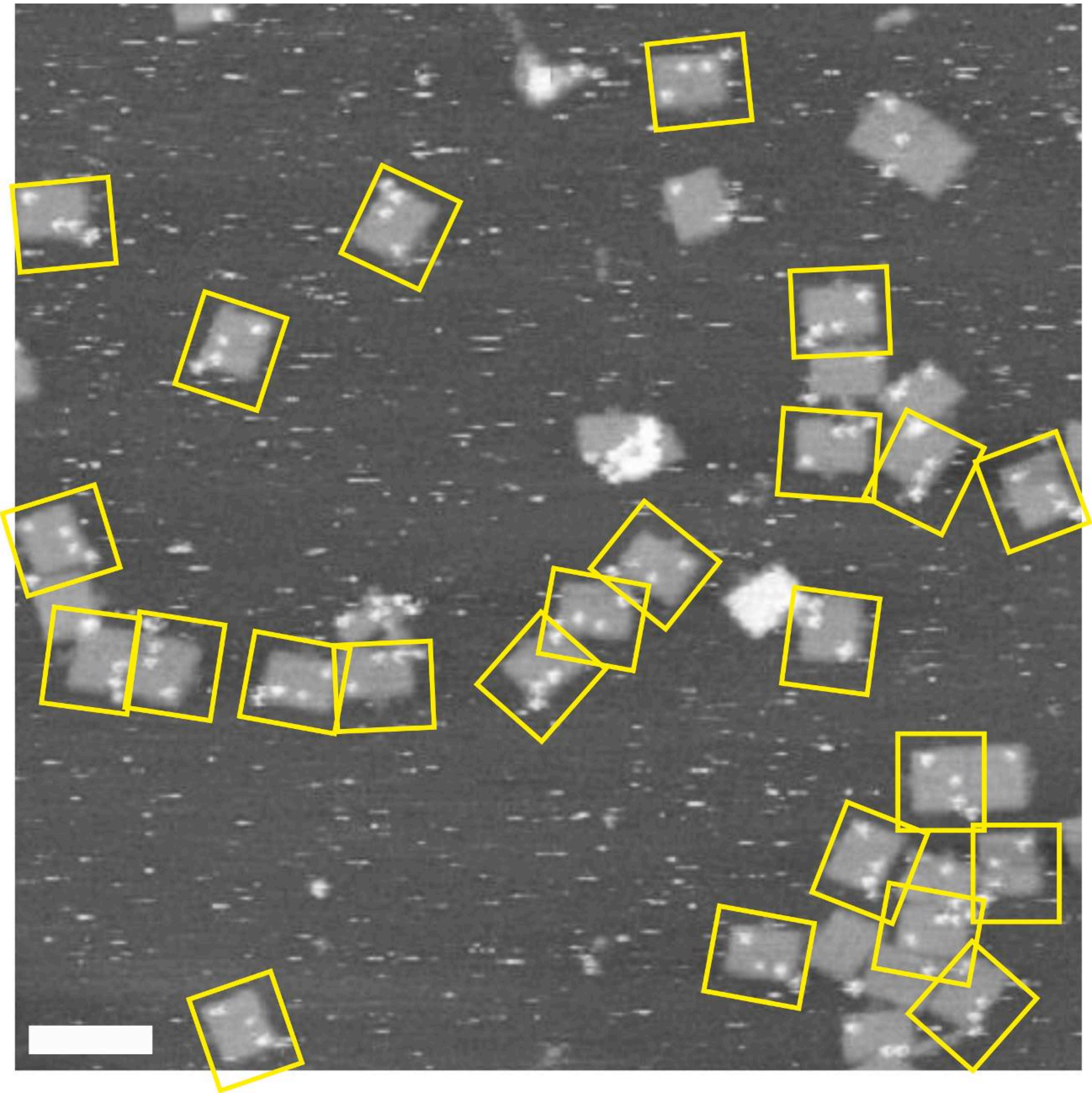

Supplementary Figure 9 | Encrypted "9, 2nd" on target rectangle DNA origami. Scale bar: 200 nm.

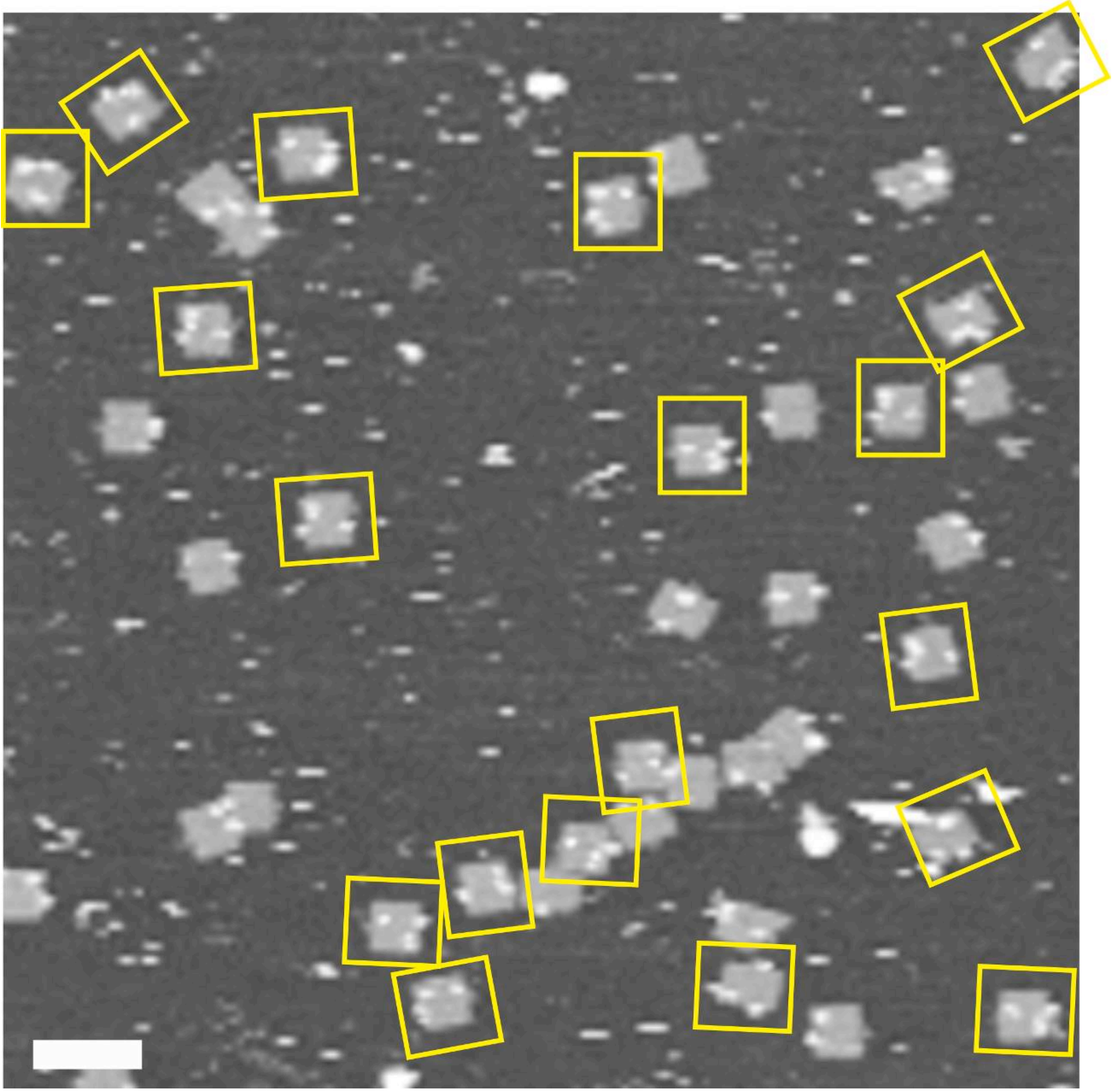

Supplementary Figure 10 | Encrypted "9, 2nd" on scaffold-sliding rectangle DNA origami. Scale bar: 200 nm.

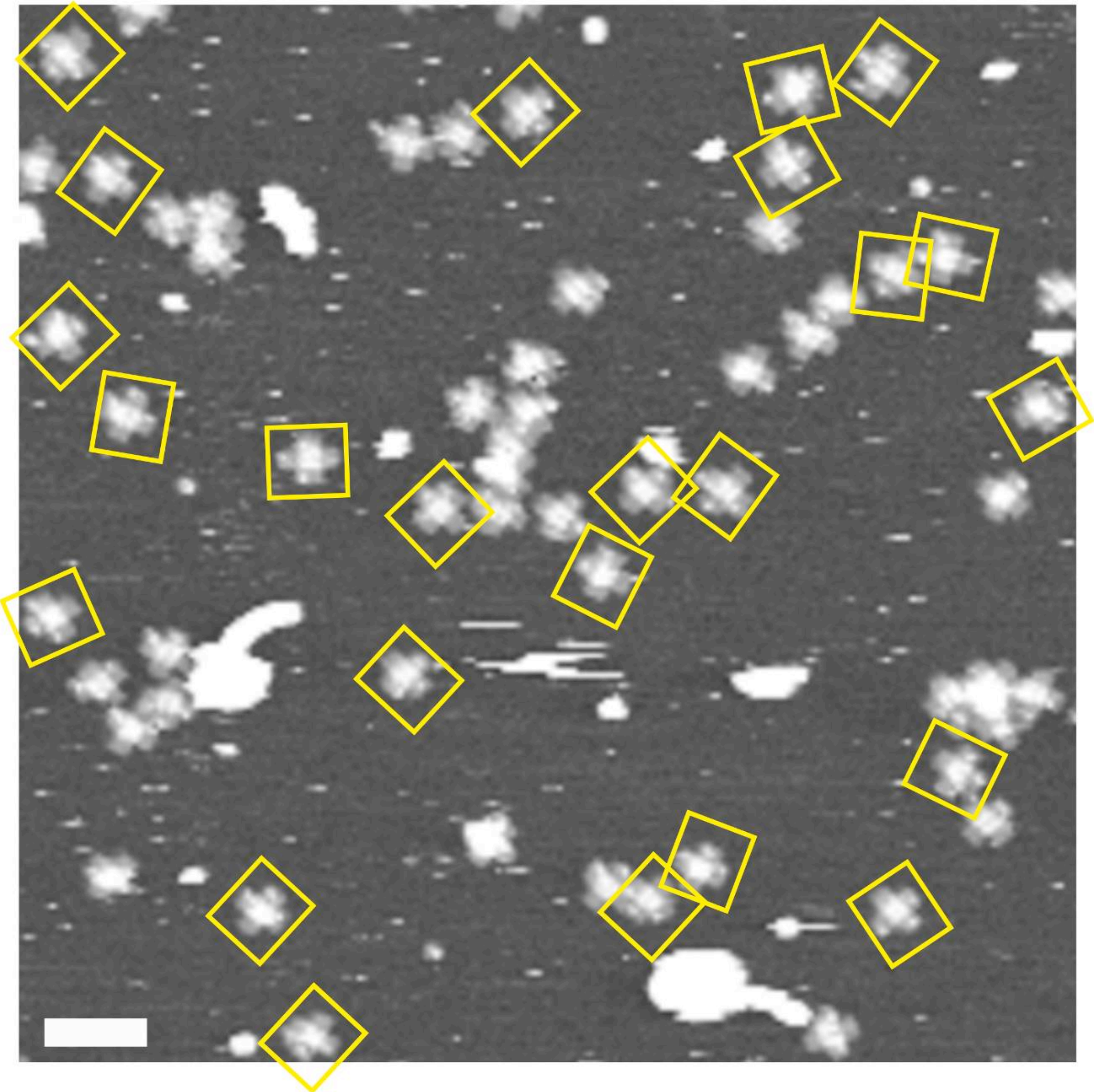

Supplementary Figure 11 | Encrypted "9, 2nd" on misrouted cross-shaped DNA origami. Scale bar: 200 nm.

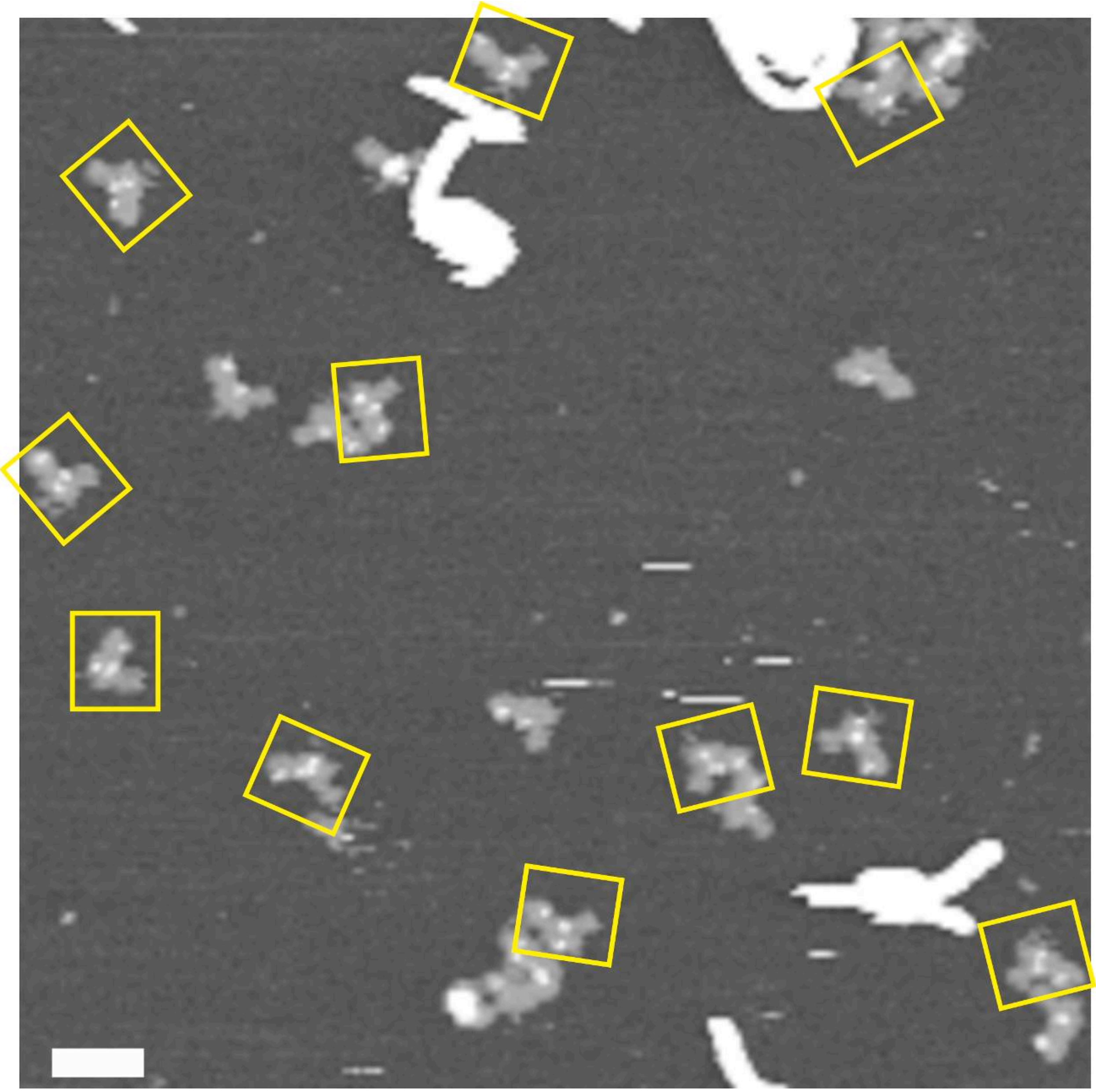

Supplementary Figure 12 | Encrypted "9, 2nd" on misrouted China map-shaped DNA origami. Scale bar: 200 nm.

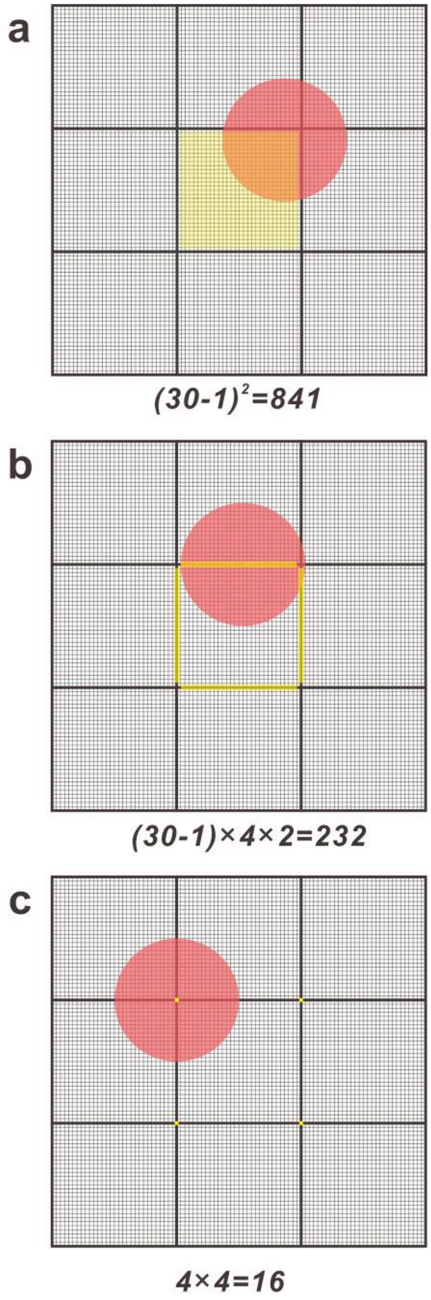

Supplementary Figure 13 | Size effect of streptavidin on the key size of DNA origami encryption. Streptavidin has a diameter of ~10 nm which is about the length of thirty base pairs. Hence, the model is built that places a 10-nm circle (red) on a sudoku made of nine 30 × 30 grids. When the circle center lies anywhere inside the yellow rectangle (a), the circle is considered to be in the grid. In this case, there are 841 situations. When the circle center moves along the four edges except the corners (b), the circle is considered to be in either of the two grids. In this case, there are 232 situations. When the circle center is at the corner (c), the circle is considered to be in one of the four grids. In this case, there are 16 situations. In total, there are 841 + 232 +16 = 1089 degenerate cases in recognizing streptavidin patterns due to its large size, which reduces the key size by only about 10 bits.

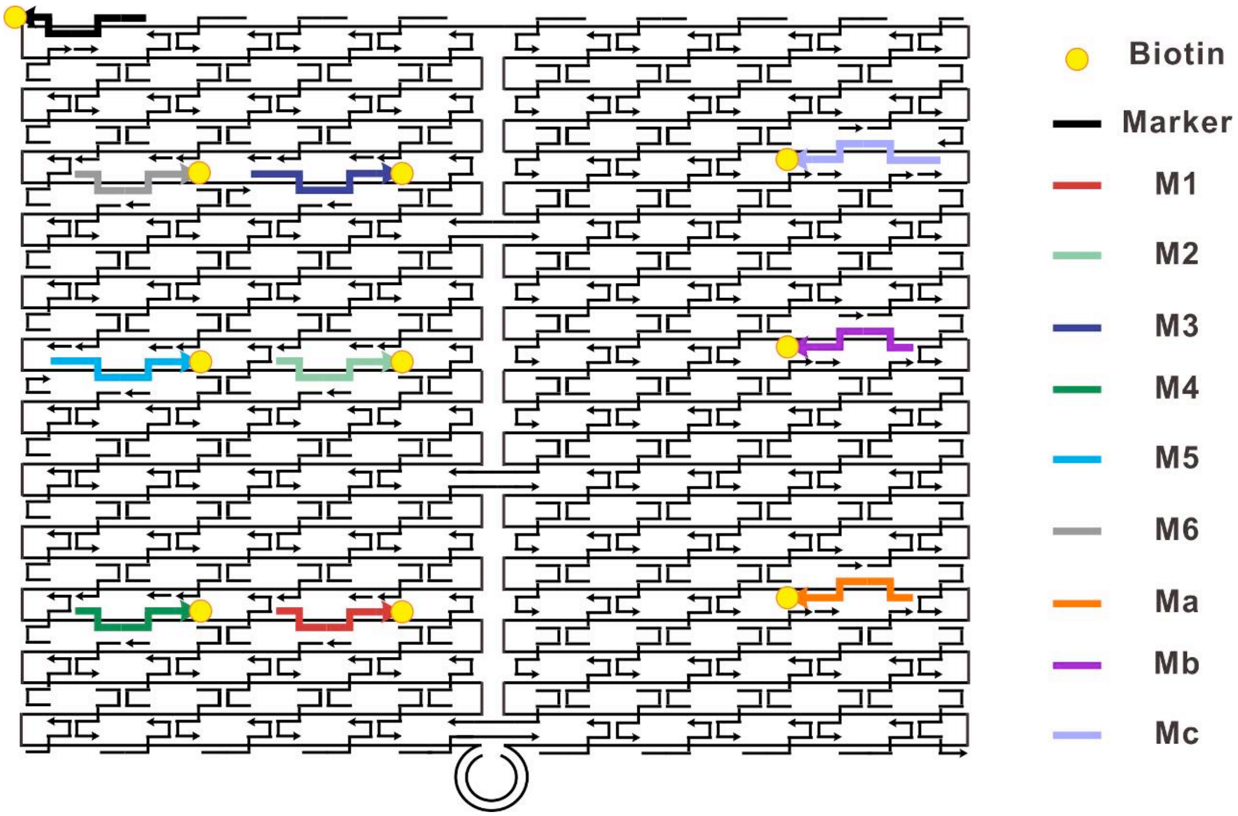

Supplementary Figure 14 | Rectangular DNA origami carrying all M-strands for eight-letter text communication. Unbound sections of staple strands are not depicted.

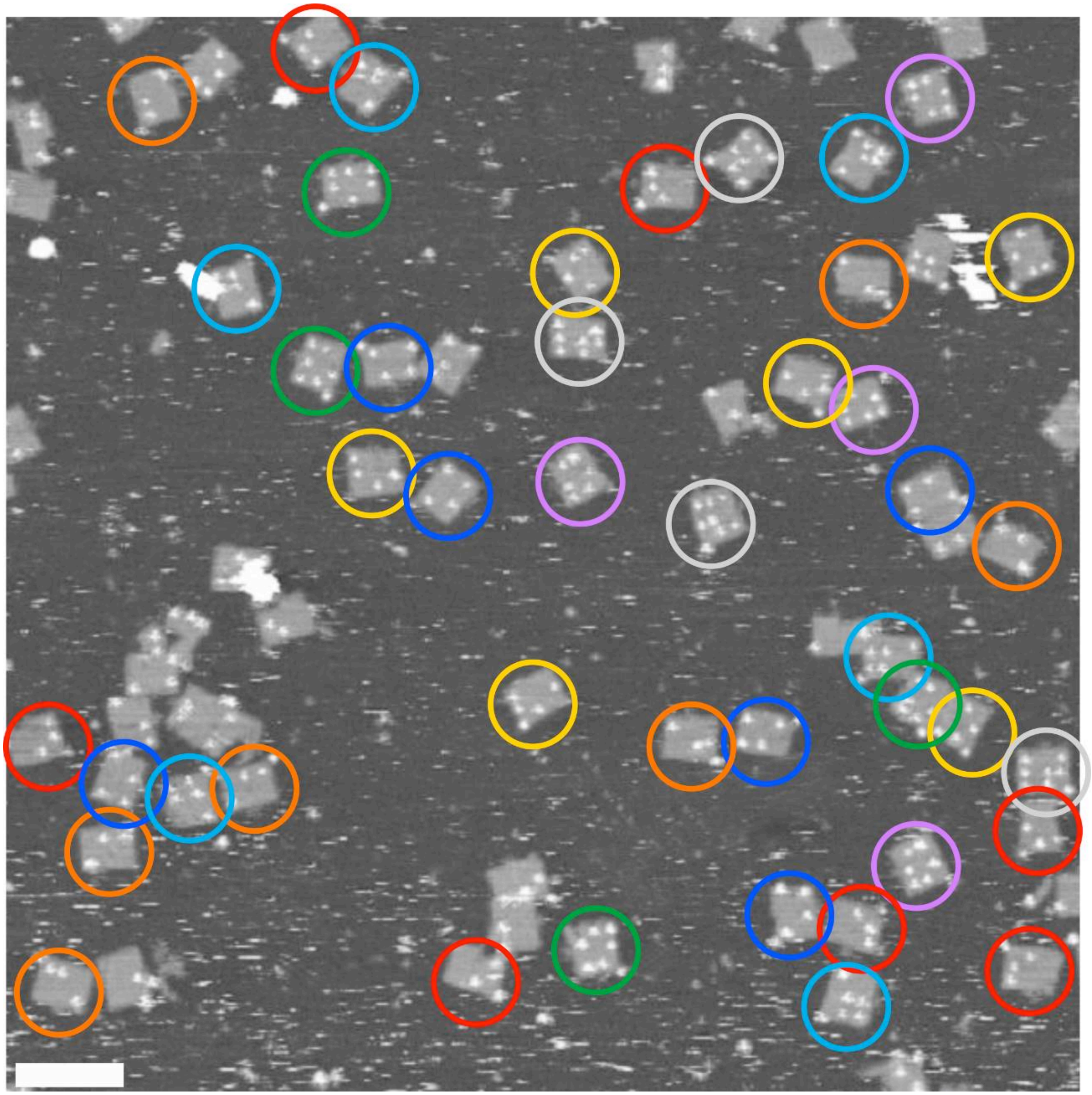

Supplementary Figure 15 | Streptavidin patterns conveying the message "19120623". Major patterns at each position are circled in the same color as in the main text. Scale bar: 200 nm.

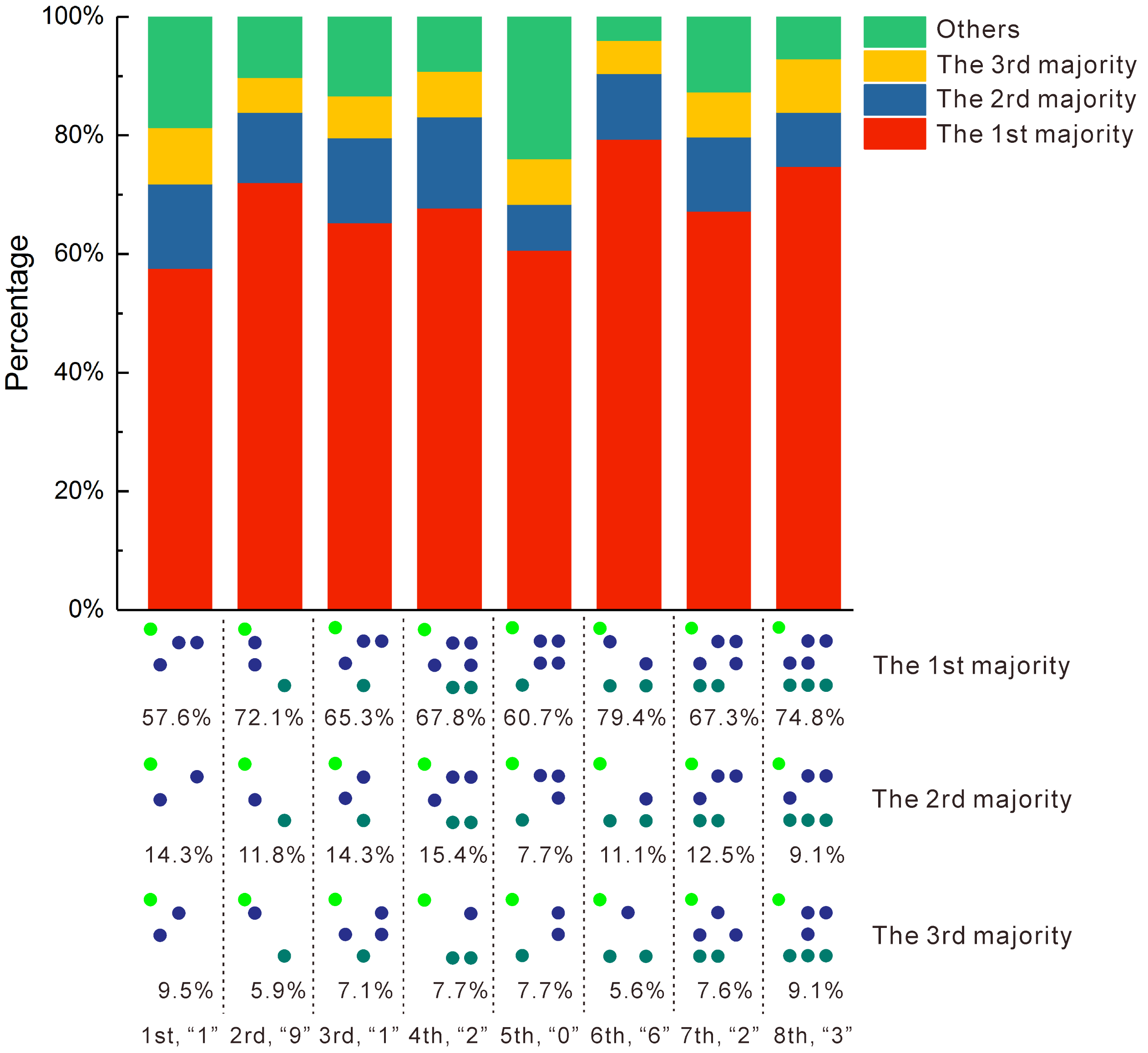

Supplementary Figure 16 | Statistics of major patterns at the eight positions conveying the message “19120623”. The top three major patterns and their percentages are demonstrated.

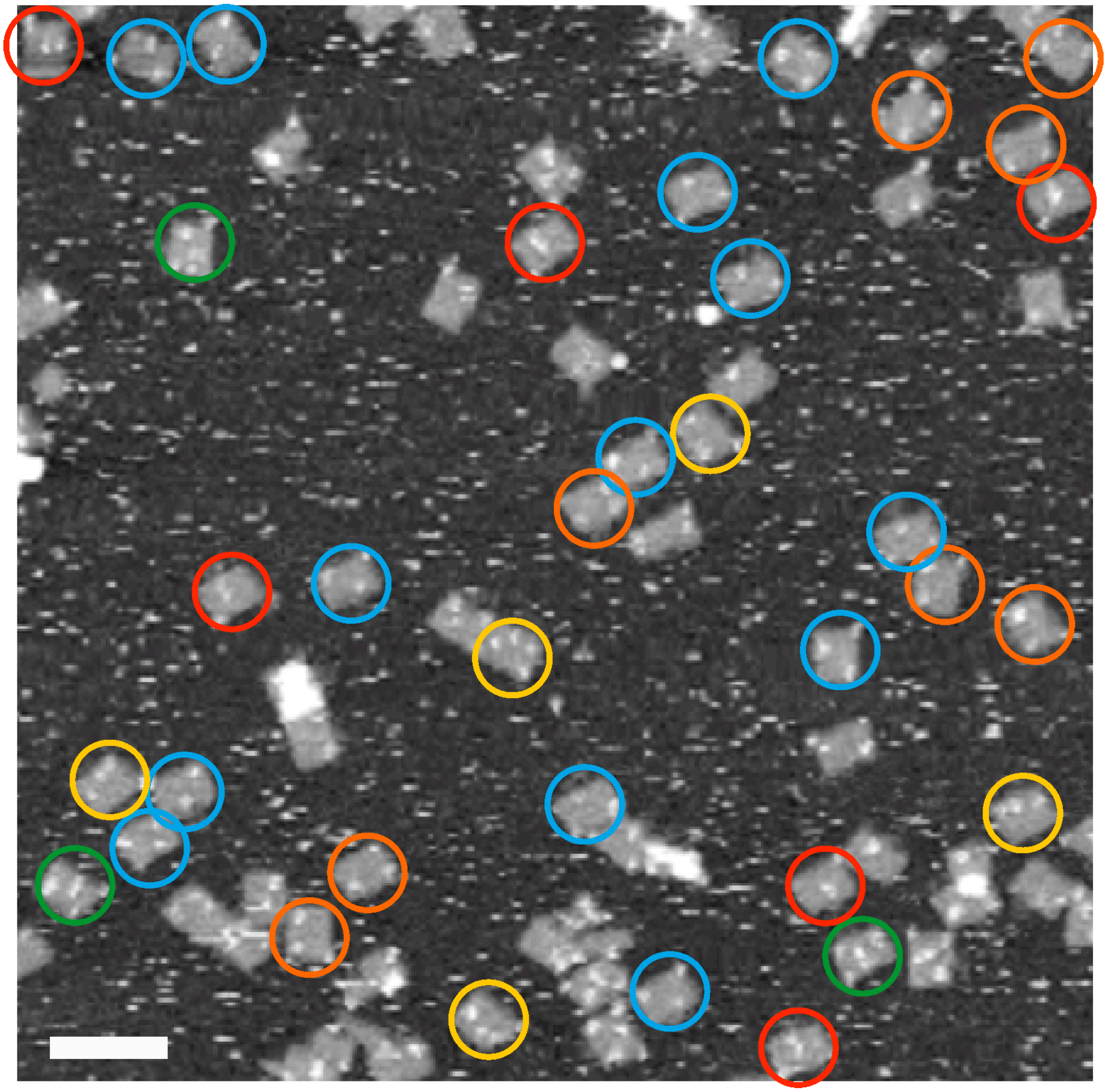

Supplementary Figure 17 | Blind test I: streptavidin patterns at the five positions conveying the message "CHINA". Major patterns at each position are circled in the same color as in the main text. Scale bar: 200 nm.

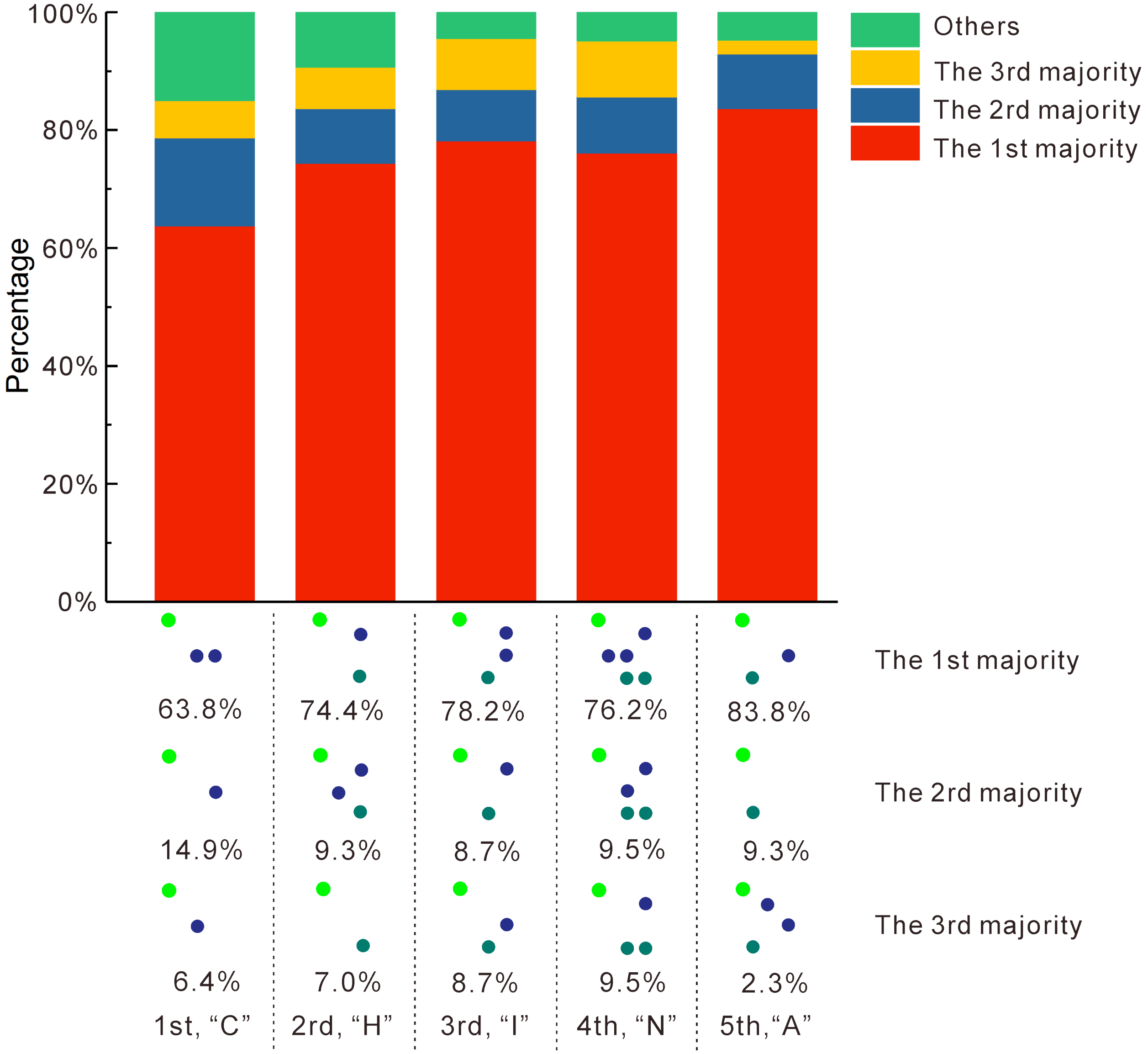

Supplementary Figure 18 | Blind test I: statistics of major patterns at the five positions conveying the message “CHINA”. The top three major patterns and their percentages are demonstrated.

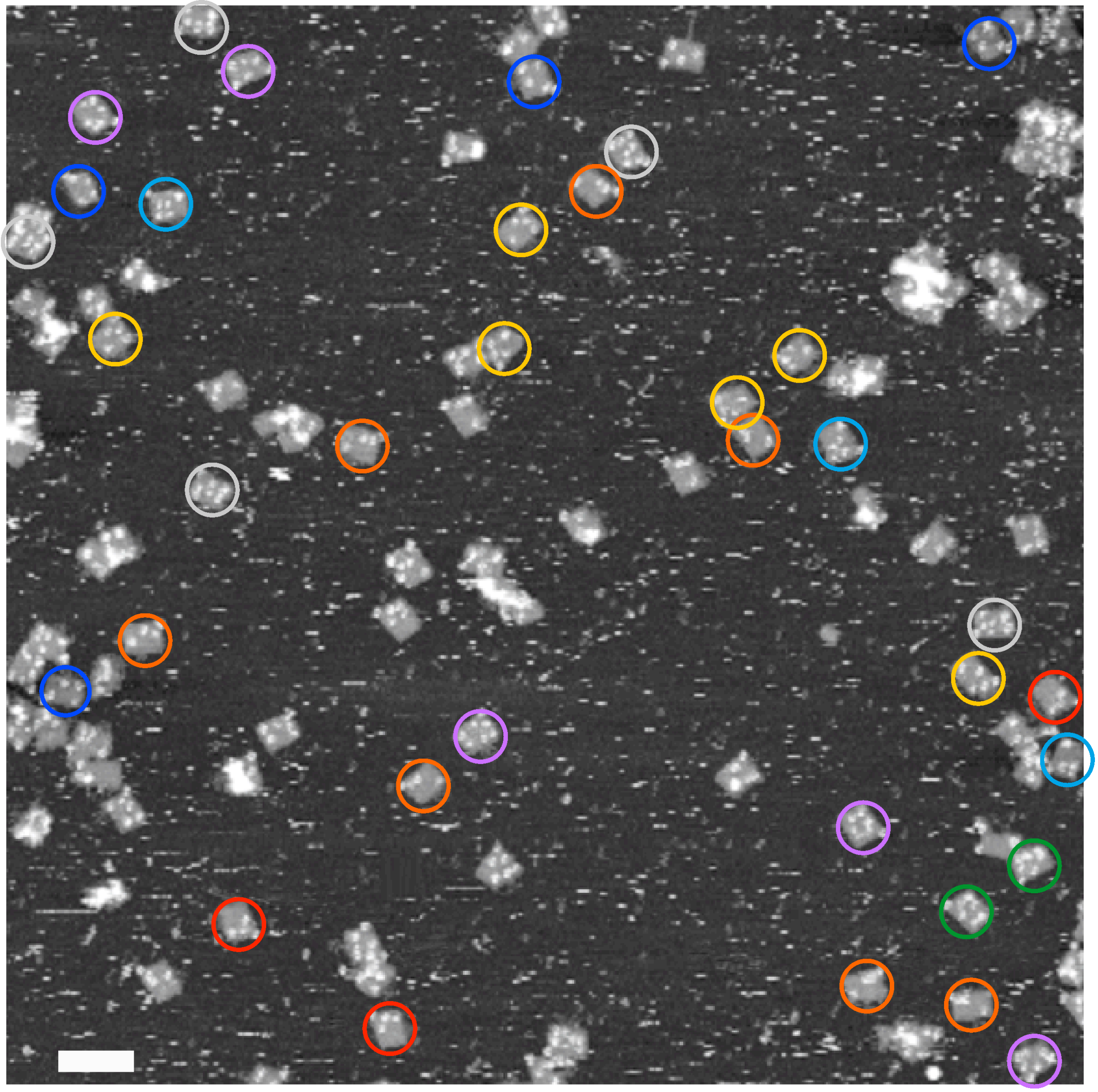

Supplementary Figure 19 | Blind test II: streptavidin patterns at the eight positions conveying the message "19120623". Major patterns at each position are circled in the same color as in the main text. Scale bar: 200 nm.

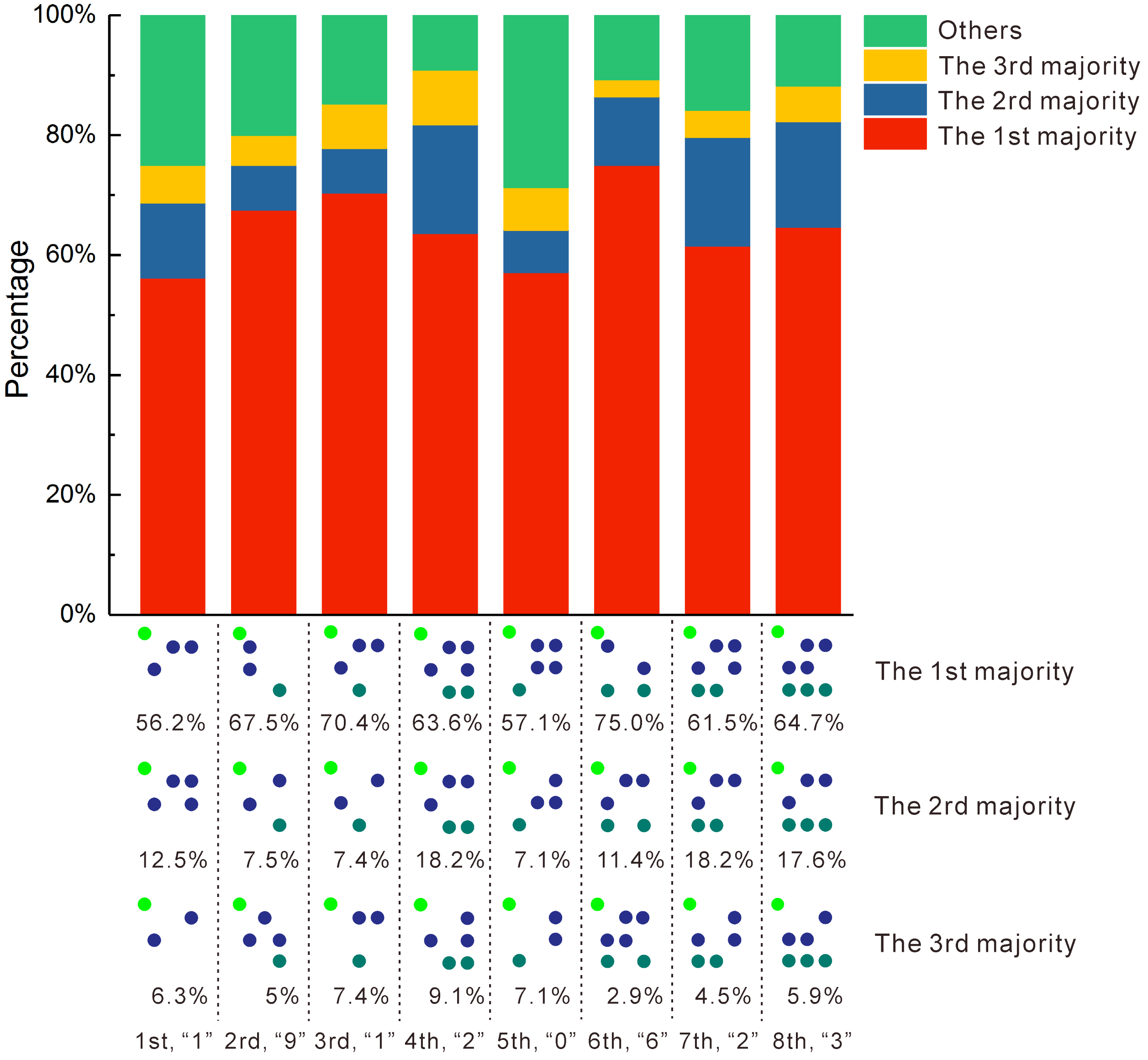

Supplementary Figure 20 | Blind test II: statistics of major patterns at the eight positions conveying the message “19120623”. The top three major patterns and their percentages are demonstrated.

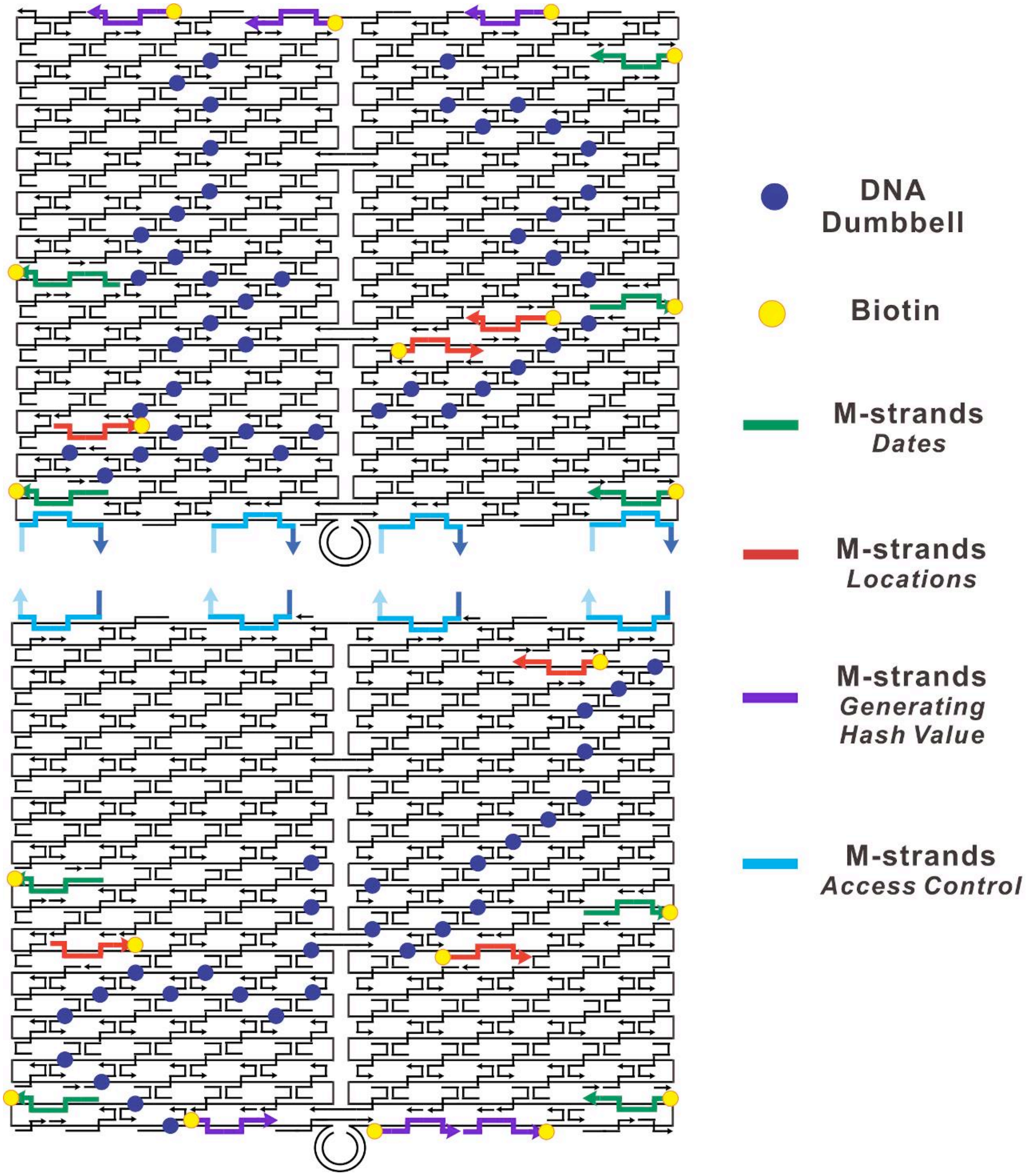

Supplementary Figure 21 | E- and F-tiles carrying all M-strands transmitting the message to Mallory. Different sticky ends are used to prevent unidirectional dimerization of E- and F-tiles. Unbound sections of staple strands are not depicted.

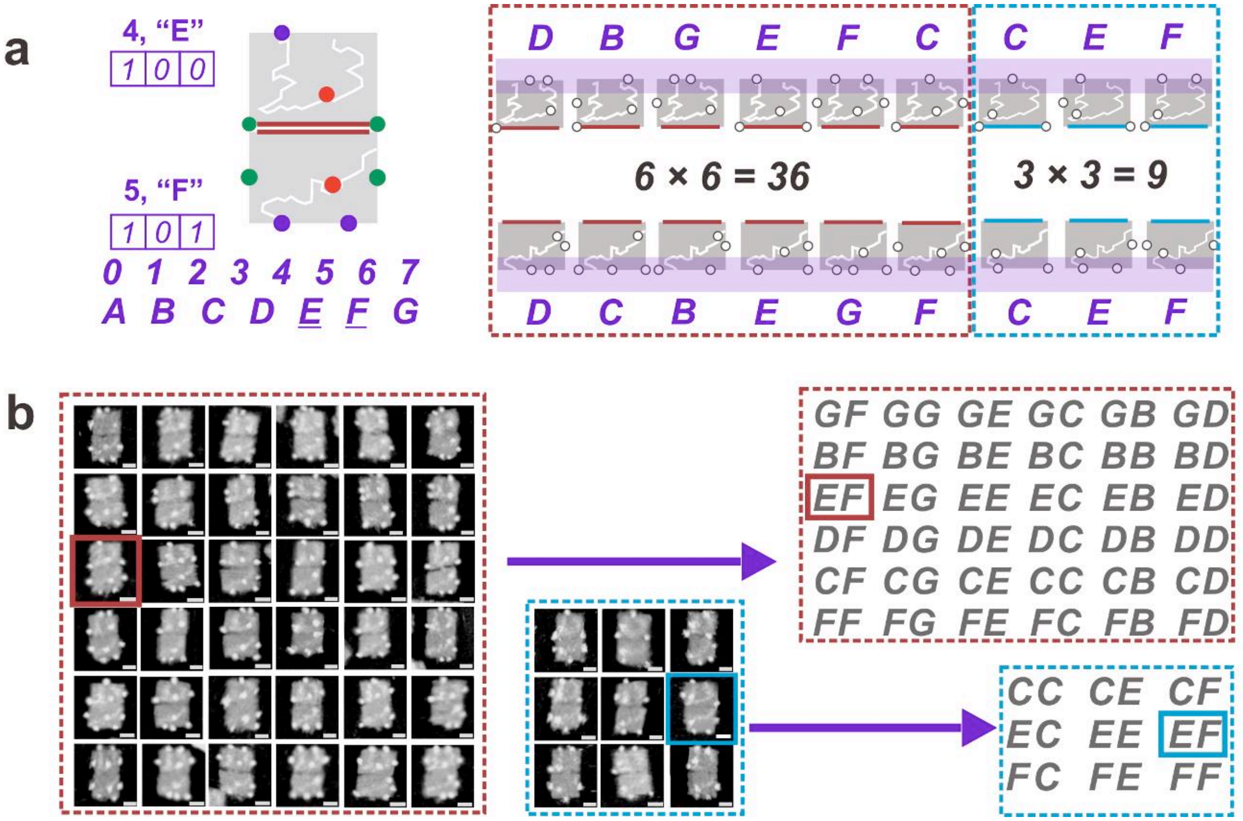

Supplementary Figure 22 | Generation of the hash value of message. (a) Representation of the hash value segment in the pattern. The purple domain at each DNA origami represents a letter that participates in generation of the hash value. (b) Generating the hash value of message from the tile dimers to Bob and Mallory, respectively. Initially, the hash value is given to them. In this case, the hash value is “GF GG GE GC GB GD BF BG BE BC BB BD EF EG EE EC EB ED DF DG DE DC DB DD CF CG CE CC CB CD FF FG FE FC FB FD” (in crimson box) or “CC CE CF EC EE EF FC FE FF” (in cyan box). Both of them are defined as correct. There is no fixed order between these double-letter segments. When Bob or Mallory obtained the folded DNA origami, they used their passwords (DNA linkers) for assembly of DNA origami dimers. Consequently, the hash value was generated from the splice of double-letter segments. In this communication, the hash value obtained with Bob’s password should be the one in crimson box as expected, while that for Mallory should be the one in cyan box. If the integrity of the message is hampered, for example, someone added misleading biotin patterns to mix with the original one, both Bob and Mallory will perceive that. That is because there are no complementary sticky ends to their DNA linkers in the misleading biotin patterns, which will result in extra unrecognizable DNA origami monomers in AFM imaging. On the contrary, if there is a lack of double-letter segments, some DNA origamis could be missing. It has been known that the pattern with the segment “EF” carried the message. Hence, both the Bob and Mallory can detect their target patterns. However, the pattern obtained by Bob is the correct one, while that found by Mallory is wrong. In the overall process, the only thing that needs to be kept secure is Bob’s password. Scale bar: 50 nm.

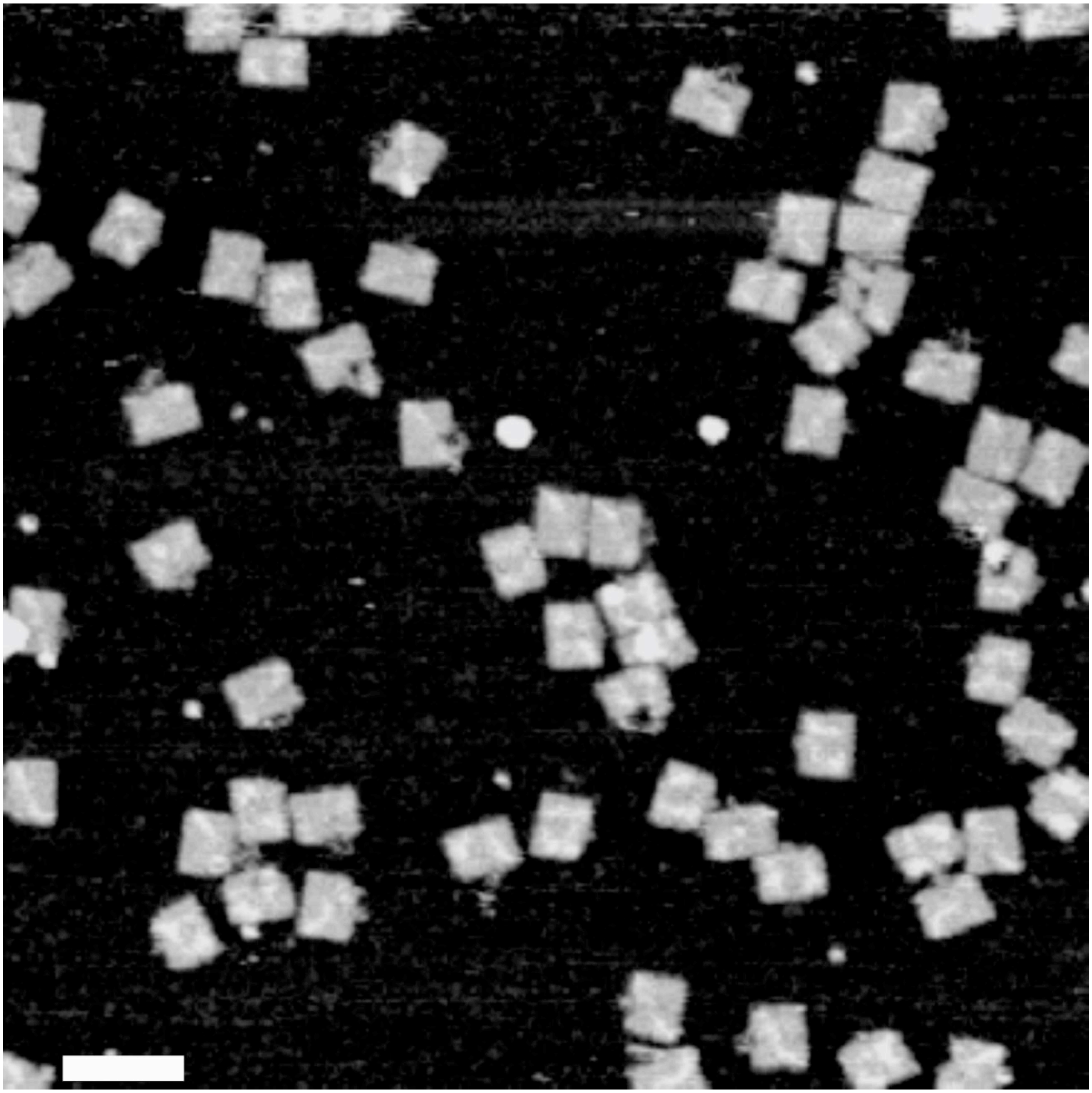

Supplementary Figure 23 | E- and F-tiles before dimerization. Scale bar: 200 nm.

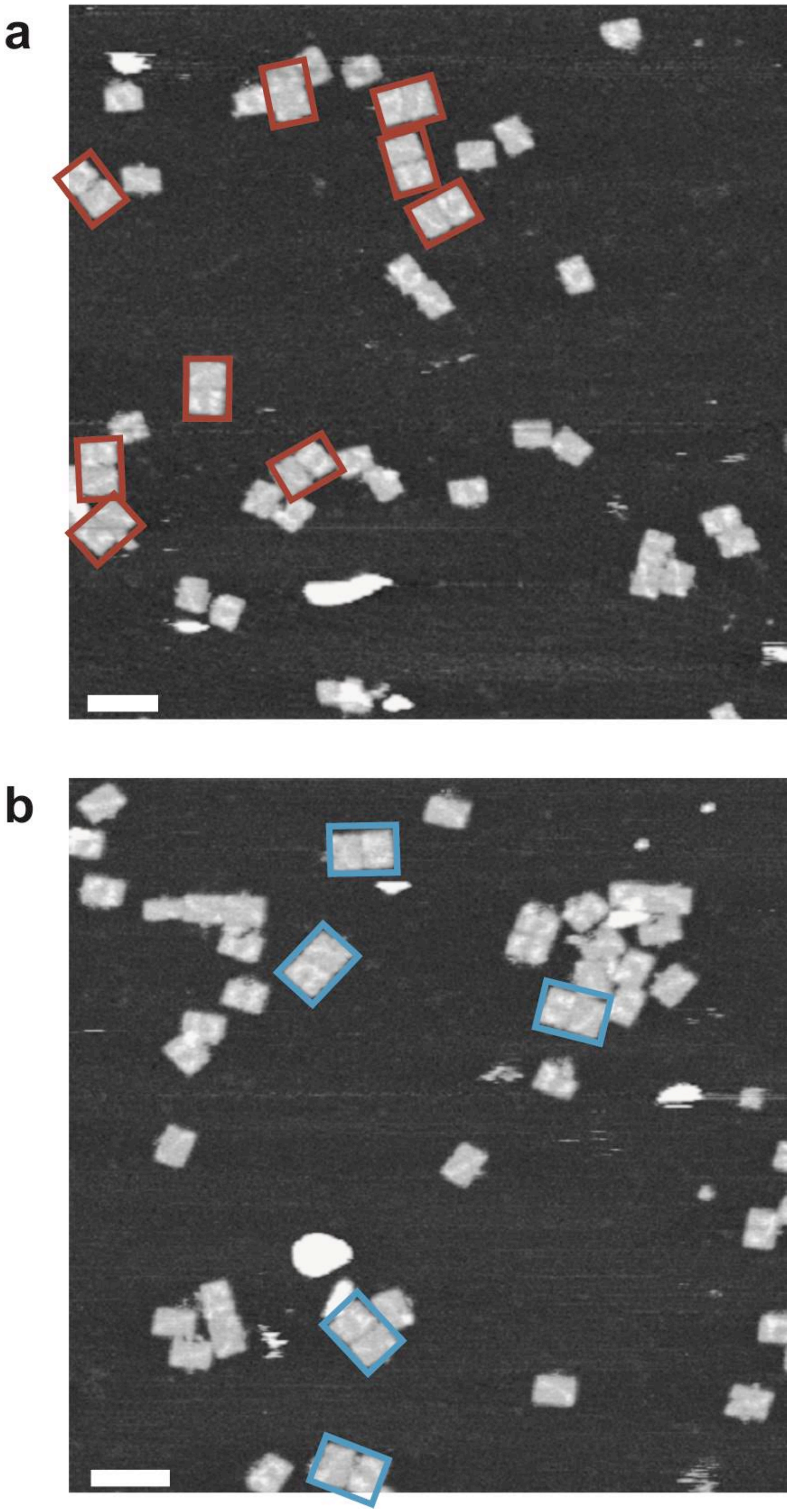

Supplementary Figure 24 | E-F tile dimers available to Bob (a) and Mallory (b), respectively. Scale bar: 200 nm.

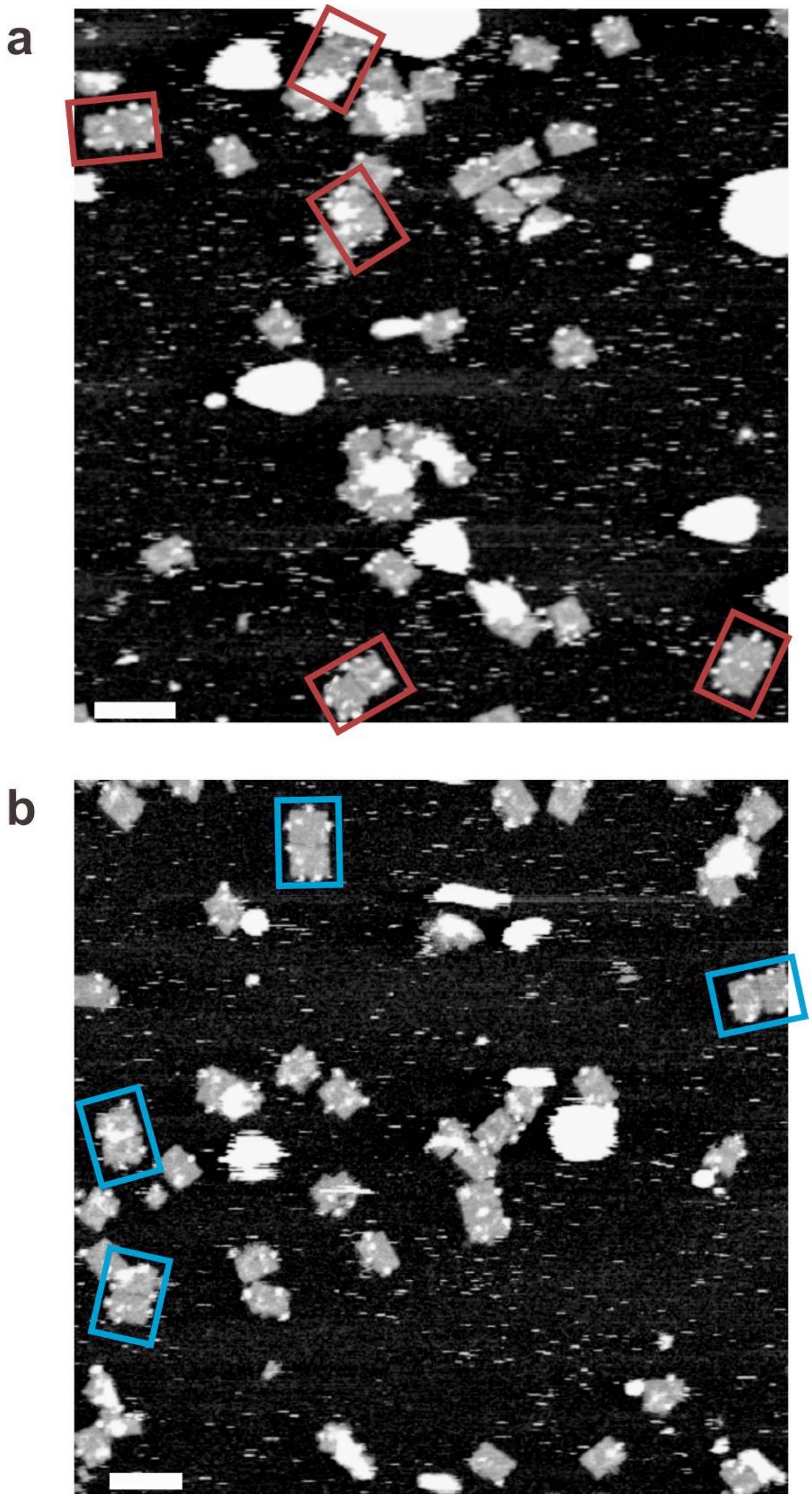

Supplementary Figure 25 | Streptavidin patterns on E-F tile dimers available to Bob (a) and Mallory (b), respectively. Scale bar: 200 nm.

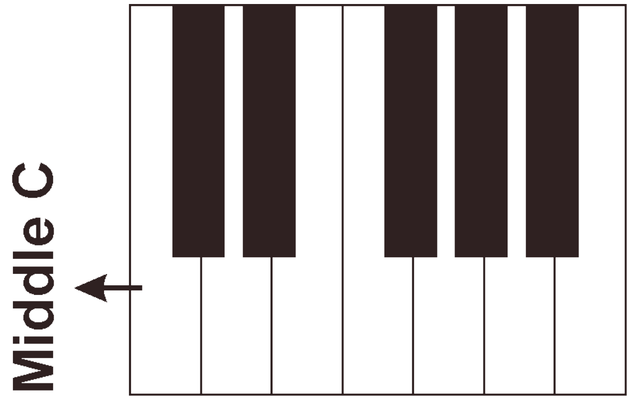

C	*0000*	G	*0100*	♯D	*1000*	•	*1100*	♪	*00*
D	*0001*	A	*0101*	♯F	*0111*	• •	*1101*	♩	*01*
E	*0010*	B	*0110*	♯G	*0001*	• • •	*1110*	𝅗𝅥	*10*
F	*0011*	♯C	*0111*	♯A	*0001*	Null	*1111*	𝅝	*11*

Supplementary Figure 26 | The piano keypad indexing notes and pitches in music to binary numbers.

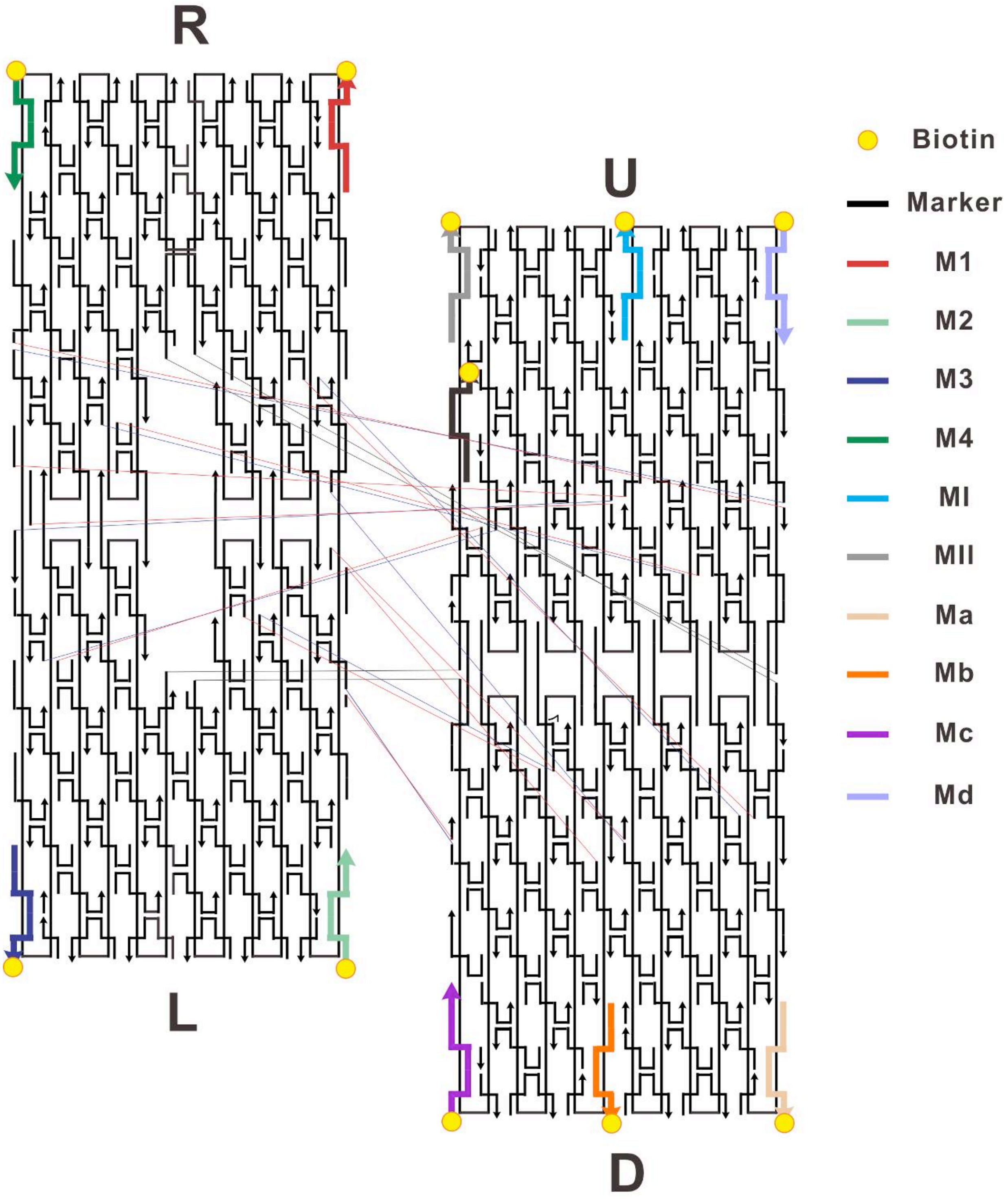

Supplementary Figure 27 | Cross-shaped DNA origami carrying all M-strands for music communication. Unbound sections of staple strands are not depicted.

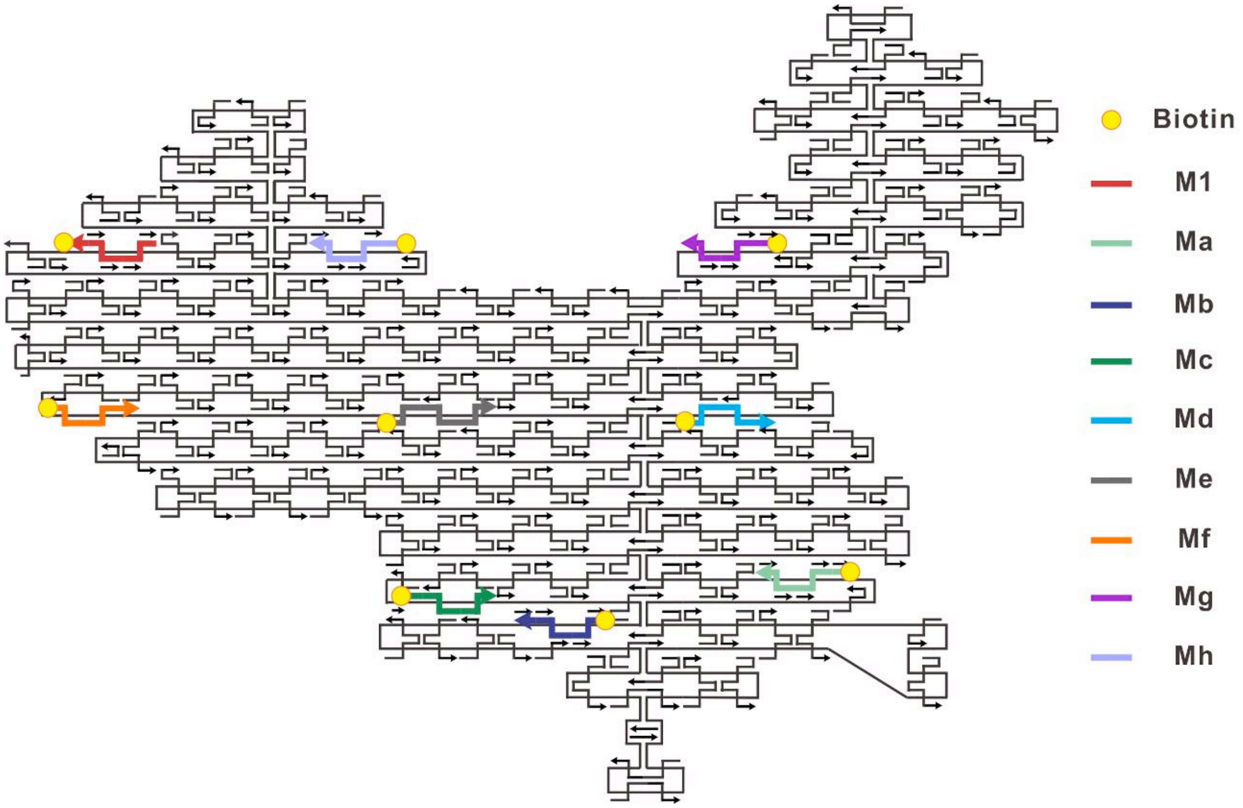

Supplementary Figure 28 | China map-shaped DNA origami carrying all M-strands for image communication. The marker is not used here since the DNA origami is asymmetric itself. Unbound sections of staple strands are not depicted.

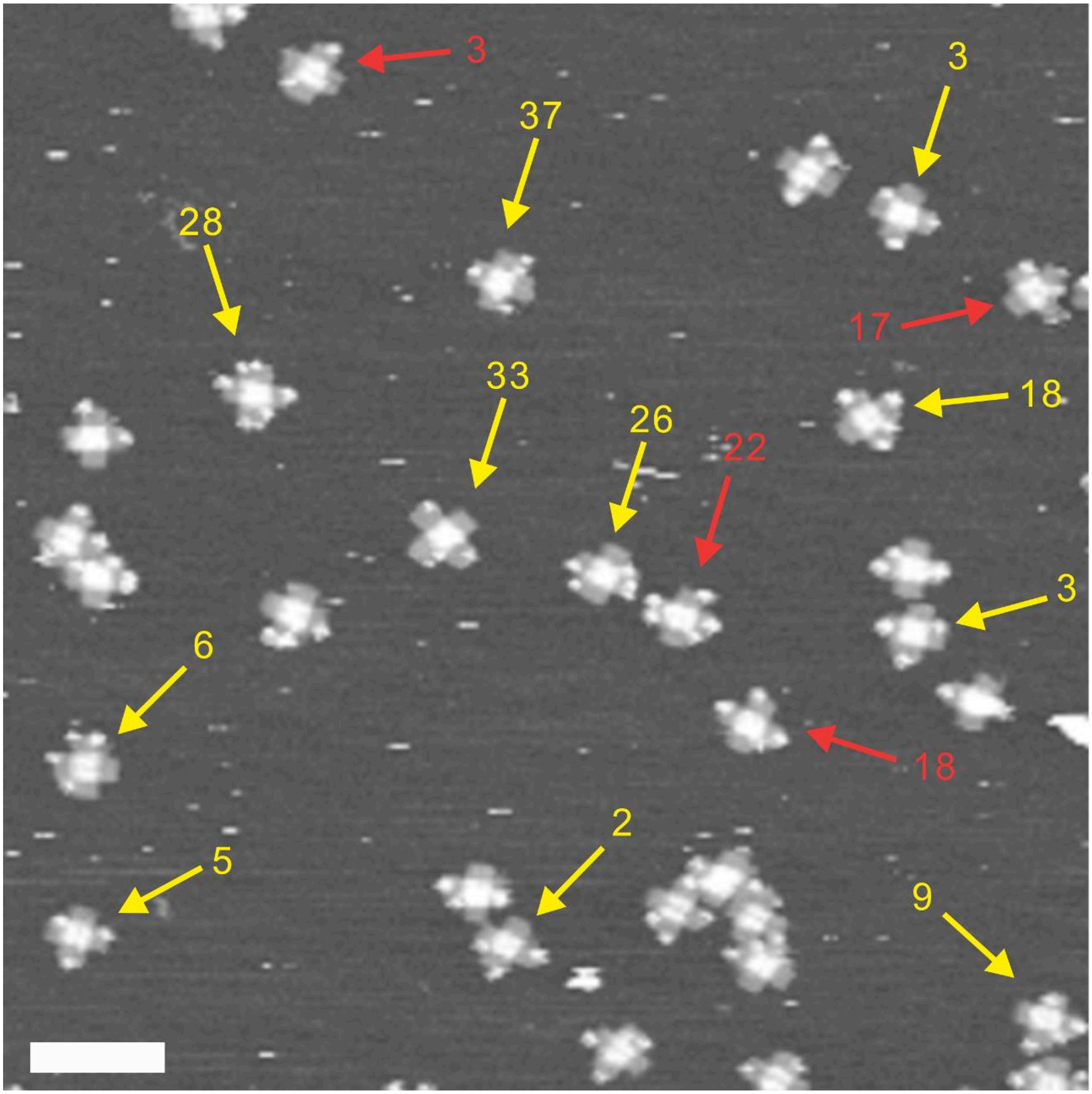

Supplementary Figure 29 | Streptavidin patterns presenting the music. Correct patterns are marked in yellow numbers denoting their positions in the music, while wrong patterns are marked in red. Scale bar: 200 nm.

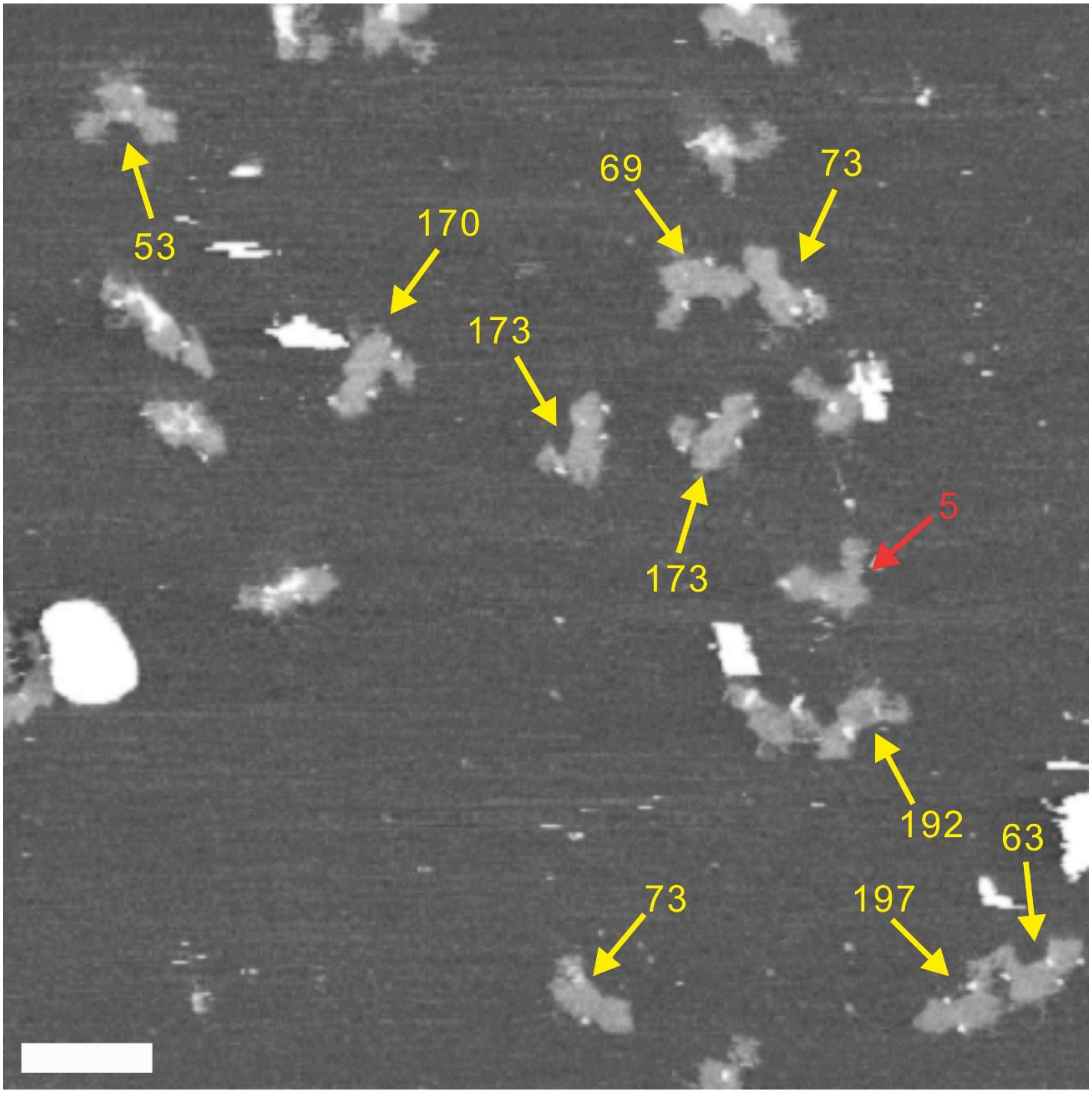

Supplementary Figure 30 | Streptavidin patterns presenting the panda image. Correct patterns are marked in yellow numbers denoting their positions in the image, while wrong patterns are marked in red. Scale bar: 200 nm.

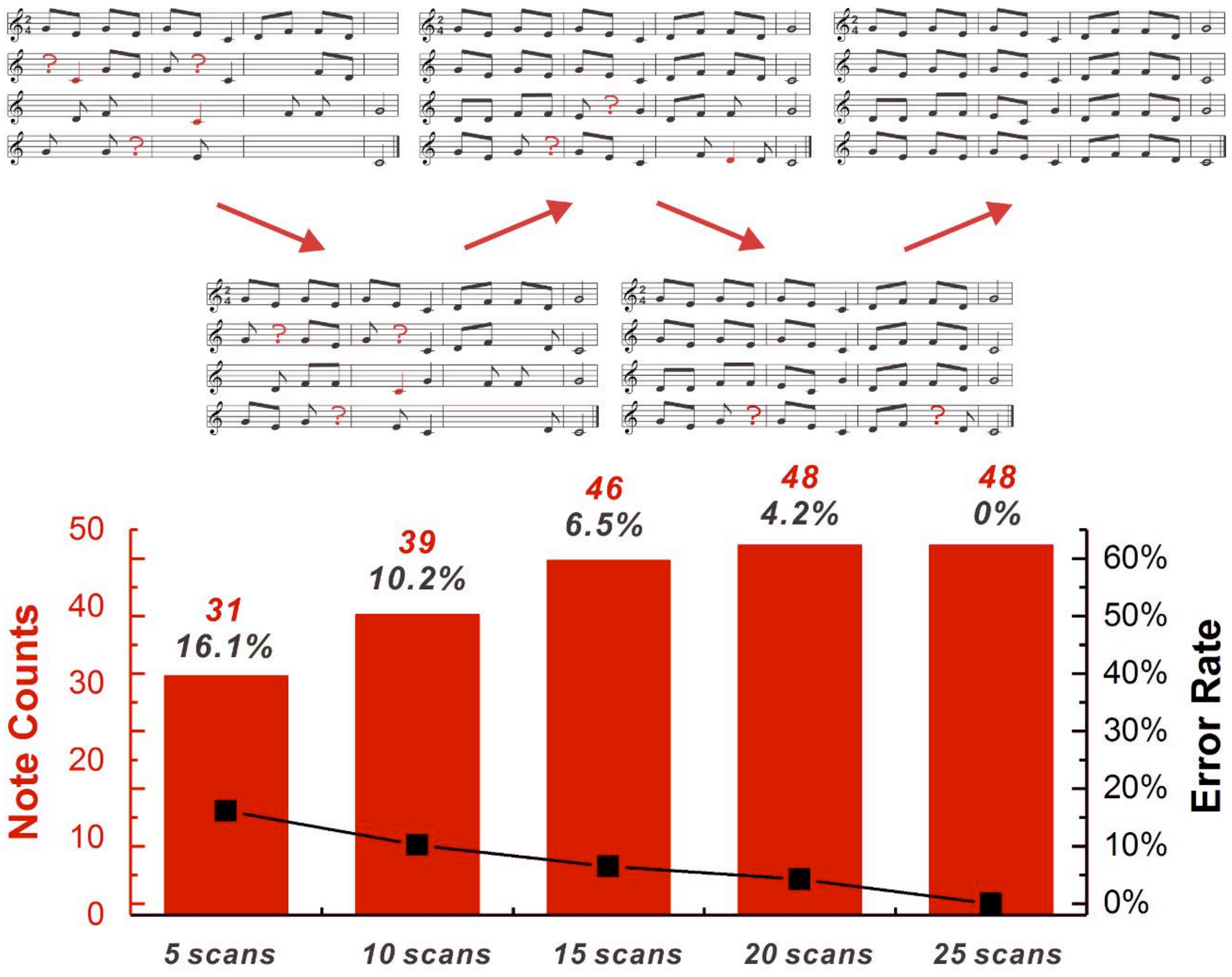

Supplementary Figure 31 | Collecting the notes of the music. Errors are denoted in red. The notes in which not a single streptavidin pattern takes the majority are given in question marks.

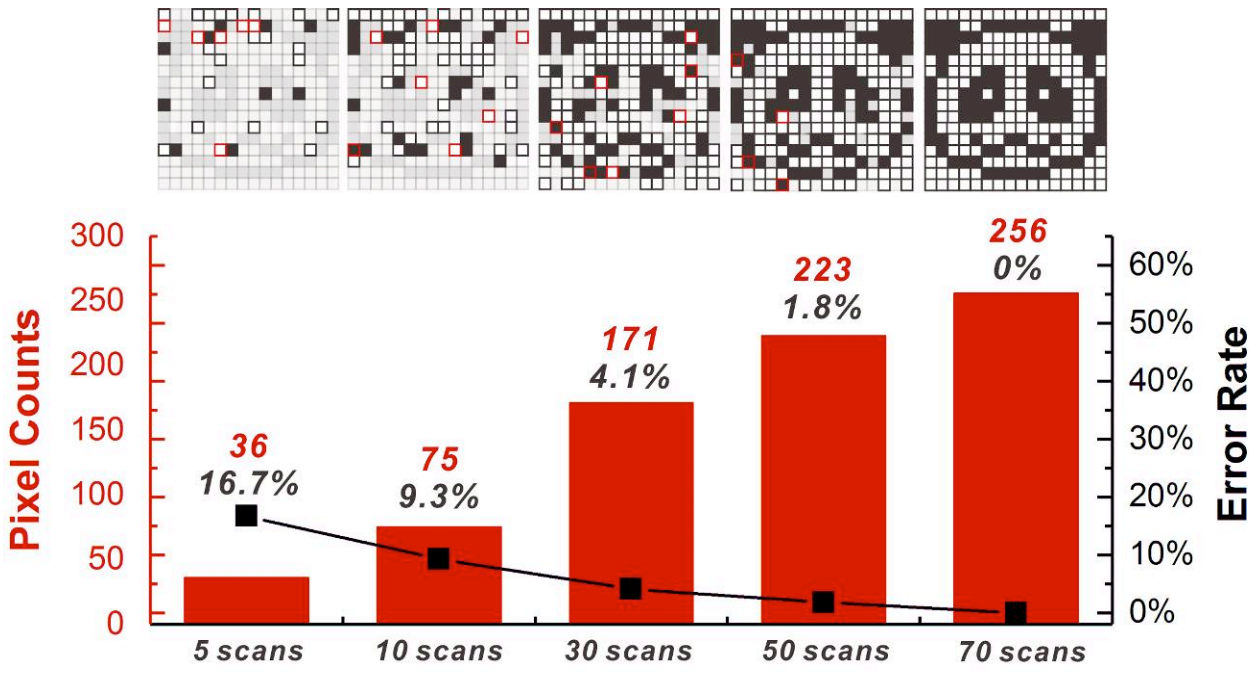

Supplementary Figure 32 | Collecting the pixels of the panda image. Errors are bracketed in red. The pixels in which not a single streptavidin pattern takes the majority are colored in white.

Supplementary Notes:
DNA Sequences
The sequence of used DNA scaffold, M13mp18, is available at: https://www.neb.com/-/media/nebus/page-images/tools-and-resources/interactive-tools/dna-sequences-and-maps/text-documents/m13mp18gbk.txt?la=en
Sequences of staple strands of rectangular, cross-shaped and China map-shaped DNA origami are given in Ref. 28, 35 and 36 in the main text, respectively.
A counterclockwise sliding of 176 nucleotides of the scaffold in rectangular DNA origami (Fig.1e) induced variation of the staple strands.

In simulation of disassociation of M-strands from the scaffold:
A-48:
GAACCGAACTGACCAACTTTGAAAGAGGACAGATGAACGGTGTACAGA
A-40: CTGACCAACTTTGAAAGAGGACAGATGAACGGTGTACAGA
A-32: CTTTGAAAGAGGACAGATGAACGGTGTACAGA
B-48: GACGGCCAGTGCCAAGCTTGCATGCCTGCAGGTCGACTCTAGAGGATC
B-40: GTGCCAAGCTTGCATGCCTGCAGGTCGACTCTAGAGGATC
B-32: CTTGCATGCCTGCAGGTCGACTCTAGAGGATC
C-48: GCCCGTATATCAAGTTTGCCTTTAGCGTCAGACTGTAGCGCGTTTTCA
C-40: ATCAAGTTTGCCTTTAGCGTCAGACTGTAGCGCGTTTTCA
C-32: TGCCTTTAGCGTCAGACTGTAGCGCGTTTTCA
D-48: CGAAGCCCTGATTGTTTGGATTATACTTCTGAATAATGGAAGGGTTAG
D-40: TGATTGTTTGGATTATACTTCTGAATAATGGAAGGGTTAG
D-32: TGGATTATACTTCTGAATAATGGAAGGGTTAG

In the fluorescent pattern:
Marker:
ACGTTAGTAAATGAATTTTCTGTATGGGATTTTGCTAAACttGTCGCTCTCTCAAGTAG
AAT
M1:
AATCATGGTCATAGCTGTTTCCTGTGTGAAATTGTTATCCttGTCGCTCTCTCAAGTAG
AAT
Fluorescent strand: ATTCTACTTGAGAGAGCGAC-Alexa 647

In the eight-digit text communication:
Marker: ACGTTAGTAAATGAATTTTCTGTATGGGATTTTGCTAAACttt
M1: AATCATGGTCATAGCTGTTTCCTGTGTGAAATTGTTATCCttt
M2: TCTGCGAACGAGTAGATTTAGTTTGACCATTAGATACATTttt
M3: GCTGACCTTCATCAAGAGTAATCTTGACAAGAACCGGATATTCATTACttt
M4: GTGCCAAGCTTGCATGCCTGCAGGTCGACTCTAGAGGATCttt
M5: CAACATGTTTTAAATATGCAACTAAAGTACGGTGTCTGGAAGTTTCATttt
M6: CTGACCAACTTTGAAAGAGGACAGATGAACGGTGTACAGAttt
Ma: TGATTGTTTGGATTATACTTCTGAATAATGGAAGGGTTAGttt
Mb: AAGAACGCGAGGCGTTTTAGCGAACCTCCCGACTTGCGGGttt
Mc: GCGACAGAATCAAGTTTGCCTTTAGCGTCAGACTGTAGCGCGTTTTCAttt

In the music communication:
Marker: AGACTCCTTATTACGCAGTATGTTAGCAAACGTAGAAAATttt
M1: ACCTACATTTTGACGCTCAATCGTCTGAAATGGATTATTTttt
M2: tttAGCTGATTGCCCTTCACCGCCTGGCCCTGAGAGAGTTGCA
M3: AGCTAATGCAGAACGCGCCTGTTTATCAACAATAGATAAGttt
M4: tttGTTAAATAAGAATAAACACCGGAATCATAATTACTAGAAA
MI: ATAAGTATAGCCCGGAATAGGTGTATCACCGTACTCAGGAttt
MII: AAAGGTGGCAACATATAAAAGAAACGCAAAGACACCACGGttt
Ma: GCTGGCTGACCTTCATCAAGAGTAATCTTCGACAAGAACCttt
Mb: TTGATTCCCAATTCTGCGAACGAGTAGATTTAGTTTGACCttt
Mc: tttCGTTAATATTTTGTTAAAATTCGCGTTAAATTTTTGTTAAATCAGCTC
Md: tttGAGGACTAAAGACTTTTTCATGAGGAAGTTTCCATTAAAC

In the image communication:
M1: ATAATTTTTTCACGTTGAAAATCTCCAAAAAAAAAGGCTCCttt
Ma: tttGCAAATCAACAGTTGAAAGGAATTGAGGAAGGTTATCTAA
Mb: tttTGCCCCAGCAGGCGAAAATCCTGTTTGATGGTGGTTCCGA
Mc: tttGAGGCGGTTTGCGTATTGGGCGCCAGGGTGGTTTTTCTTT
Md: tttAAATCATAGGTCTGAGAGACTACCTTTTTAACCTCCGGCT
Me: tttTACATTTCGCAAATGGTCAATAACCTGTTTAGCTATATTTTCATTTGG
Mf: tttAGCTTAATTGCTGAATATAATGCTGTAGCTCAACATGTTT
Mg: tttCACCGACTTGAGCCATTTGGGAATTAGAGCCAGCAAAATC
Mh: tttCCAGTAAGCGTCATACATGGCTTTTGATGATACAGGAGTG

In the Overload Operation communication:
M-strands for representation of dates and locations:
D1: AGAATTAGCAAAATTAAGCAATAAAGCCTCAGAGCATAAAGCTAAATCttt
D2: TGGACTCCAACGTCAAAGGGCGAAAAACCGTCTATCAGGGttt
D3: tttATAAATCCTCATTAAAGCCAGAATGGAAAGCGCAGTCTCT
D4: AGAGGCATTTTCGAGCCAGTAATAAGAGAATATAAAGTACttt
D5: tttACCAGTAATAAAAGGGACATTCTGGCCAACAGAGATAGAA

M-strands for representation of locations:
On E-tiles:
E-L1: GTGCCAAGCTTGCATGCCTGCAGGTCGACTCTAGAGGATCttt
E-L2: tttCTGAGAAGAGTCAATAGTGAATTTATCAAAATCATAGGTC
E-L3: tttAATGGTTTGAAATACCGACCGTGTGATAAATAAGGCGTTA
On F-tiles:
F-L1: AAAACTAGCATGTCAATCATATGTACCCCGGTTGATAATCttt
F-L2: tttATCAAAATCATAGGTCTGAGAGACTACCTTTTTAACCTCC
F-L3: tttCAGTCTCTGAATTTACCGTTCCAGTAAGCGTCATACATGG

M-strands for generation of hash value:
On E-tiles:
E-H1: tttCGTAACGATCTAAAGTTTTGTCGTCTTTCCAGACGTTAGT
E-H2: tttCGTAACACTGAGTTTCGTCACCAGTACAAACTACAACGCC

E-H3: tttCCCTCAGAACCGCCACCCTCAGAACCGCCACCCTCAGAGC
On F-tiles:
F-H1: tttAAGGGAGCCCCCGATTTAGAGCTTGACGGGGAAAGCCGGC
F-H2: tttCGGCCTTGCTGGTAATATCCAGAACAATATTACCGCCAGC
F-H3: CATTGCAACAGGAAAAACGCTCATGGAAATACCTACATTTttt

M-strands containing sticky ends for Bob:
On E-tiles:
E-B1:
GCGAATGTtttCGATGGCCCACTACGTGAACCATCACCCAAATCAAGTTTTtttGTCGCT
CT
E-B2:
GCGAATGTtttCCCCGATTTAGAGCTTGACGGGGAAAGCCGGCGAACGTGGtttGTCGCT
CT
E-B3:
GCGAATGTtttCGGCCTTGCTGGTAATATCCAGAACAATATTACCGCCAGCtttGTCGCT
CT
E-B4:
GCGAATGTtttGAAATGGATTATTTACATTGGCAGATTCACCAGTCACACGtttGTCGCT
CT
On F-tiles:
F-B1:
GTCGCTCTtttACGTTAGTAAATGAATTTTCTGTATGGGATTTTGCTAAACtttGCGAATG
T
F-B2:
GTCGCTCTtttGTCACCAGTACAAACTACAACGCCTGTAGCATTCCACAGAtttGCGAAT
GT
F-B3:
GTCGCTCTtttCACCACCCTCATTTTCAGGGATAGCAAGCCCAATAGGAACtttGCGAAT
GT
F-B4:
GTCGCTCTtttTATAAGTATAGCCCGGAATAGGTGTATCACCGTACTCAGGtttGCGAAT
GT

M-strands containing sticky ends for Mallory:
On E-tiles:
E-M1:
ATGTCCGCtttCGATGGCCCACTACGTGAACCATCACCCAAATCAAGTTTTtttAGAGCA
TC
E-M2:
ATGTCCGCtttCCCCGATTTAGAGCTTGACGGGGAAAGCCGGCGAACGTGGtttAGAGC
ATC
E-M3:
ATGTCCGCtttCGGCCTTGCTGGTAATATCCAGAACAATATTACCGCCAGCtttAGAGCA
TC

E-M4:
ATGTCCGCtttGAAATGGATTATTTACATTGGCAGATTCACCAGTCACACGtttAGAGCA
TC
On F-tiles:
F-M1:
AGAGCATCtttACGTTAGTAAATGAATTTTCTGTATGGGATTTTGCTAAACtttATGTCCG
C
F-M2:
AGAGCATCtttGTCACCAGTACAAACTACAACGCCTGTAGCATTCCACAGAtttATGTCC
GC
F-M3:
AGAGCATCtttCACCACCCTCATTTTCAGGGATAGCAAGCCCAATAGGAACtttATGTCC
GC
F-M4:
AGAGCATCtttTATAAGTATAGCCCGGAATAGGTGTATCACCGTACTCAGGtttATGTCC
GC

Bob’s password:
BP1: AGAGCGACAGAGCGAC
BP2: ACATTCGCACATTCGC

Mallory’s password:
MP1: GATGCTCTGATGCTCT
MP2: GCGGACATGCGGACAT

M-strands for representation of the England and France maps:
Map1:
AGGGTTGAATAAATCCTCCTCttttGAGGAACAAGttttCTTGTTCATTAAATGATATTC
Map2:
GCATAAAGTTCCACACTCCTCttttGAGGAACAAGttttCTTGTAACATACGAAGCGCCA
Map3:
GCTCACAATGTAAAGCTCCTCttttGAGGAACAAGttttCTTGTCTGGGGTGGGTTTGCC
Map4:
TTCGCCATTGCCGGAATCCTCttttGAGGAACAAGttttCTTGTACCAGGCATTAAATCA
Map5:
GCTTCTGGTCAGGCTGTCCTCttttGAGGAACAAGttttCTTGTCGCAACTGTGTTATCC
Map6:
AGACAGTCATTCAAAATCCTCttttGAGGAACAAGttttCTTGTGGGTGAGAAGCTATAT
Map7:
TTTCATTTGGTCAATATCCTCttttGAGGAACAAGttttCTTGTACCTGTTTATATCGCG
Map8:
TCGCAAATGGGGCGCGTCCTCttttGAGGAACAAGttttCTTGTAGCTGAAATAATGTGT
Map9:
ATCGGCTGCGAGCATGTCCTCttttGAGGAACAAGttttCTTGTTAGAAACCTATCATAT
Map10:
GTGAGCTAGTTTCCTGTCCTCttttGAGGAACAAGttttCTTGTTGTGAAATTTGGGAAG

Map11:
TCATAGCTACTCACATTCCTCttttGAGGAACAAGttttCTTGTTAATTGCGCCCTGAGA
Map12:
GGCGATCGCACTCCAGTCCTCttttGAGGAACAAGttttCTTGTCCAGCTTTGCCATCAA
Map13:
AAATAATTTTAAATTGTCCTCttttGAGGAACAAGttttCTTGTTAAACGTTGATATTCA
Map14:
GCAAATATCGCGTCTGTCCTCttttGAGGAACAAGttttCTTGTGCCTTCCTGGCCTCAG
Map15:
ACCGTTCTAAATGCAATCCTCttttGAGGAACAAGttttCTTGTTGCCTGAGAGGTGGCA
Map16:
TATATTTTAGCTGATATCCTCttttGAGGAACAAGttttCTTGTAATTAATGTTGTATAA
Map17:
CGAGTAGAACTAATAGTCCTCttttGAGGAACAAGttttCTTGTTAGTAGCAAACCCTCA
Map18:
CATTCAACGCGAGAGGTCCTCttttGAGGAACAAGttttCTTGTCTTTTGCATATTATAG
Map19:
GCGTTATAGAAAAAGCTCCTCttttGAGGAACAAGttttCTTGTCTGTTTAGAAGGCCGG
Map20:
AGTAATCTTAAATTGGTCCTCttttGAGGAACAAGttttCTTGTGCTTGAGAGAATACCA
Map21:
ATACGTAAAAGTACAATCCTCttttGAGGAACAAGttttCTTGTCGGAGATTTCATCAAG
Map22:
AAAAAAGGACAACCATTCCTCttttGAGGAACAAGttttCTTGTCGCCCACGCGGGTAAA
Map23:
GTAAAGCACTAAATCGTCCTCttttGAGGAACAAGttttCTTGTGAACCCTAGTTGTTCC
Map24:
AGCTGATTACAAGAGTTCCTCttttGAGGAACAAGttttCTTGTCCACTATTGAGGTGCC
Map25:
GCTCATTTTCGCATTATCCTCttttGAGGAACAAGttttCTTGTAATTTTTGAGCTTAGA
Map26:
ACTGCCCGCCGAGCTCTCCTCttttGAGGAACAAGttttCTTGTGAATTCGTTATTACGC
Map27:
CCCGGGTACTTTCCAGTCCTCttttGAGGAACAAGttttCTTGTTCGGGAAACGGGCAAC
Map28:
CAGCTGGCGGACGACGTCCTCttttGAGGAACAAGttttCTTGTACAGTATCGTAGCCAG
Map29:
GTTTGAGGGAAAGGGGTCCTCttttGAGGAACAAGttttCTTGTGATGTGCTAGAGGATC
Map30:
CTTTCATCCCCAAAAATCCTCttttGAGGAACAAGttttCTTGTCAGGAAGACCGGAGAG
Map31:
AGAAAAGCAACATTAATCCTCttttGAGGAACAAGttttCTTGTATGTGAGCATCTGCCA
Map32:
CAATAAATACAGTTGATCCTCttttGAGGAACAAGttttCTTGTTTCCCAATTTAGAGAG
Map33:
TCCATATACATACAGGTCCTCttttGAGGAACAAGttttCTTGTCAAGGCAACTTTATTT

Map34:
TACCTTTAAGGTCTTTTCCTCttttGAGGAACAAGttttCTTGTACCCTGACAAAGAAGT
Map35:
CAAAAATCATTGCTCCTCCTCttttGAGGAACAAGttttCTTGTTTTTGATAAGTTTCAT
Map36:
CGCCTGATGGAAGTTTTCCTCttttGAGGAACAAGttttCTTGTCCATTAAACATAACCG
Map37:
TGGACTCCCTTTTCACTCCTCttttGAGGAACAAGttttCTTGTCAGTGAGACCTGTCGT
Map38:
GCCAGCTGCCTGCAGGTCCTCttttGAGGAACAAGttttCTTGTTCGACTCTGCAAGGCG
Map39:
CTTGCATGCATTAATGTCCTCttttGAGGAACAAGttttCTTGTAATCGGCCCGCCAGGG
Map40:
ATTAAGTTCGCATCGTTCCTCttttGAGGAACAAGttttCTTGTAACCGTGCGAGTAACA
Map41:
TAGATGGGGGGTAACGTCCTCttttGAGGAACAAGttttCTTGTCCAGGGTTGTGCCAAG
Map42:
CTTTTACACAGATGAATCCTCttttGAGGAACAAGttttCTTGTTATACAGTAAACAATT
Map43:
TTTAACGTTCGGGAGATCCTCttttGAGGAACAAGttttCTTGTAACAATAATTTTCCCT
Map44:
CTGAAACAGGTAATAATCCTCttttGAGGAACAAGttttCTTGTGTTTTAACCCCTCAGA
Map45:
GCCACCACTCTTTTCATCCTCttttGAGGAACAAGttttCTTGTTAATCAAACCGTCACC
Map46:
AGCGCCAACCATTTGGTCCTCttttGAGGAACAAGttttCTTGTGAATTAGATTATTAGC
Map47:
TAAGTCCTACCAAGTATCCTCttttGAGGAACAAGttttCTTGTCCGCACTCTTAGTTGC
Map48:
ACGCTCAAAATAAGAATCCTCttttGAGGAACAAGttttCTTGTTAAACACCGTGAATTT
Map49:
AGGCGTTACAGTAGGGTCCTCttttGAGGAACAAGttttCTTGTCTTAATTGACAATAGA
Map50:
CCTGATTGAAAGAAATTCCTCttttGAGGAACAAGttttCTTGTTGCGTAGACCCGAACG
Map51:
ACAGAAATCTTTGAATTCCTCttttGAGGAACAAGttttCTTGTACCAAGTTCCTTGCTT
Map52:
CACCAGAGTTCGGTCATCCTCttttGAGGAACAAGttttCTTGTTAGCCCCCGCCAGCAA
Map53:
TCACAATCGTAGCACCTCCTCttttGAGGAACAAGttttCTTGTATTACCATCGTTTTCA
Map54:
TTTTGTTTAAGCCTTATCCTCttttGAGGAACAAGttttCTTGTAATCAAGAATCGAGAA
Map55:
AGGTTTTGAACGTCAATCCTCttttGAGGAACAAGttttCTTGTAAATGAAAGCGCTAAT
Map56:
AATGCAGACCGTTTTTTCCTCttttGAGGAACAAGttttCTTGTATTTTCATCTTGCGGG

Map57:
TAACCTCCATATGTGATCCTCttttGAGGAACAAGttttCTTGTGTGAATAAACAAAATC
Map58:
AAATCAATGGCTTAGGTCCTCttttGAGGAACAAGttttCTTGTTTGGGTTACTAAATTT
Map59:
TGAGGCAGGCGTCAGATCCTCttttGAGGAACAAGttttCTTGTCTGTAGCGTAGCAAGG
Map60:
TGCCTTTAGTCAGACGTCCTCttttGAGGAACAAGttttCTTGTATTGGCCTGCCAGAAT
Map61:
CCGGAAACACACCACGTCCTCttttGAGGAACAAGttttCTTGTGAATAAGTAAGACTCC
Map62:
TTATTACGGTCAGAGGTCCTCttttGAGGAACAAGttttCTTGTGTAATTGAATAGCAGC
Map63:
TCATTACCCGACAATATCCTCttttGAGGAACAAGttttCTTGTAACAACATATTTAGGC
Map64:
AGAGGCATAATTTCATTCCTCttttGAGGAACAAGttttCTTGTCTTCTGACTATAACTA

Supplementary References

1. D. Grothues, C. R. Cantor, C. L. Smith, PCR amplification of megabase DNA with tagged random primers (T-PCR). *Nucleic Acids Res.* **21**, 1321-1322 (1993).
2. N. Zou, S. Ditty, B. Li, S. C. Lo, Random priming PCR strategy to amplify and clone trace amounts of DNA. *Biotechniques* **35**, 758-760, 762-755 (2003).
3. Wong, N. Y., Xing, H., Tan, L. H. & Lu, Y. Nano-encrypted Morse code: a versatile approach to programmable and reversible nanoscale assembly and disassembly. *J. Am. Chem. Soc.* **135**, 2931-2934 (2013).
